# Self-Powered Triboelectric Biofluid Sensors for Early Disease Screening and Diagnosis: A Review

**DOI:** 10.3390/nano16171117

**Published:** 2026-09-04

**Authors:** Yanqin Zhang, Huawen Wen, Jianbing Huang, Qiliang Zhu

**Affiliations:** 1 School of Petrochemical Engineering, Guangzhou Institute of Technology, Guangzhou 510725, China; wen_vince@hotmail.com (H.W.); huangjianbing13@163.com (J.H.); 2 Collaborative Innovation Center of Henan Province for Energy-Saving Building Materials, Xinyang Normal University, Xinyang 464000, China; 3 Center for Green Innovation, School of Mathematics and Physics, University of Science and Technology Beijing, Beijing 100083, China

**Keywords:** triboelectric, sensors, biofluids, self-powered, disease screening

## Abstract

With the growing demand for continuous health assessment and early disease screening, biofluids have attracted increasing attention because they provide molecular information that cannot be obtained from conventional physical signals alone. However, practical biofluid analysis remains constrained by limited sample volumes, unstable wet interfaces, complex fluid transport, and dependence on external power sources. Triboelectric nanogenerators and triboelectric nanosensors offer a promising solution by combining mechanical energy harvesting with direct signal transduction. This review provides a critical overview of the theoretical basis of triboelectric biofluid sensing, including working modes, figures of merit, charge-transfer mechanisms, and the differences between solid–solid and solid–liquid electrification. Material selection, interfacial functionalization, fluid collection, wearable configurations, and multimodal integration are further discussed. Recent applications involving sweat, tears, saliva, urine, interstitial fluid, blood, and wound exudate are summarized to clarify how triboelectric devices function as either power sources or active sensing interfaces. Particular attention is given to their potential in early screening, risk assessment, and auxiliary diagnosis. Current limitations associated with biofouling, charge dissipation, individual variability, biosafety, durability, calibration, and clinical validation are also evaluated. Finally, future directions are proposed to improve analytical reliability and accelerate the translation of self-powered biofluid sensors from laboratory prototypes to clinically meaningful healthcare platforms.

## 1. Introduction

With the rapid development of personalized medicine, digital health, and telemedicine, continuous health monitoring has become an important component of modern healthcare [1,2,3]. Most diagnostic procedures still rely on measurements collected at discrete time points in hospitals or laboratories. Although effective for confirming established disease, this approach may miss short-lived physiological changes that occur during early or subclinical stages [4]. Wearable devices offer a partial solution by tracking heart rate, electrocardiographic signals, body temperature, respiration, and movement over extended periods [5,6,7,8,9]. These measurements, however, primarily describe physiological function or behavior and do not directly reveal many biochemical changes associated with metabolic disturbance, inflammation, infection, or endocrine dysregulation [10,11]. Battery dependence presents another practical limitation, particularly for prolonged use, because it increases device volume and maintenance requirements while raising concerns over service life, safety, and electronic waste.

Biofluids provide a complementary source of molecular information that is more directly associated with physiological and pathological processes [12,13]. Sweat and saliva offer convenient routes for noninvasive sampling, whereas tears reflect changes in the ocular microenvironment [14]. Urine is particularly valuable for renal and metabolic screening, while interstitial fluid supports continuous assessment of metabolites and electrolytes [15]. Wound exudate contains local information on inflammation, infection, and tissue repair [16]. Blood remains the most information-rich clinical biofluid, but invasive collection and dependence on trained personnel limit its suitability for frequent, continuous, or home-based testing [17,18]. Biofluid analysis therefore provides an important bridge between conventional clinical assays and wearable health monitoring, supporting early screening, risk assessment, and longitudinal disease management.

The presence of diagnostically relevant biomarkers alone, however, does not ensure reliable sensing. Biofluids are often available only in limited volumes, while their composition, secretion rate, pH, ionic strength, and other physicochemical properties vary considerably across individuals and sampling conditions [19,20]. During collection and transport, samples may also be affected by evaporation, environmental contamination, mechanical disturbance, and nonspecific adsorption of proteins or other constituents, which can cause interfacial fouling and signal drift [21,22,23]. A practical biofluid sensing platform must therefore integrate sample collection, controlled fluid transport, molecular recognition, signal transduction, and data readout in a coordinated manner [24,25,26]. Although electrochemical, optical, and colorimetric methods have achieved high analytical sensitivity [27], many existing systems still depend on external power sources, relatively complex electronic hardware, or multistep signal-processing procedures [28,29]. Achieving reliable in situ biofluid analysis using soft, lightweight, and energy-efficient devices consequently remains a central challenge for wearable and point-of-care sensing [30].

Triboelectric nanogenerators (TENGs) and triboelectric nanosensors (TENSor) provide a distinctive strategy for addressing this challenge. Through contact electrification and electrostatic induction, they convert low-frequency mechanical inputs, including human motion, droplet impact, fluid flow, interfacial contact, and tissue deformation, into electrical energy or sensing signals [31,32,33]. Their role in biofluid analysis extends beyond replacing conventional batteries. As energy-harvesting units, TENGs can support electrochemical sensing, iontophoretic sweat stimulation, visual readout, signal processing, and wireless communication, while being integrated with microfluidic sampling and transport structures [34,35]. When used directly as sensing interfaces, their electrical outputs can reflect droplet dynamics, wetting behavior, ionic composition, molecular adsorption, and changes in interfacial charge transfer [36,37,38]. This dual functionality enables triboelectric platforms to combine energy harvesting with active biochemical or physicochemical sensing, distinguishing them from conventional wearable sensors that rely entirely on external power sources.

Over the past decade, biofluid-based triboelectric nanogenerators (BF-TENG) sensing has expanded from sweat analysis to ocular and oral monitoring, with emerging applications involving urine, interstitial fluid, blood, and wound exudate [39,40,41]. By integrating fluid management, molecular recognition, flexible electronics, and wireless readout, these platforms are shifting from the pursuit of high electrical output toward the coordinated implementation of energy harvesting, biochemical detection, and information transmission [42,43]. Practical application, however, remains constrained by the complexity of real biofluids. Ionic screening, protein adsorption, microorganisms, and cellular debris can alter interfacial charge transfer and induce signal attenuation or drift, while variations in secretion rate, sample volume, mechanical stimulation, and individual physiology complicate calibration and data interpretation [44,45,46]. Moreover, many studies still rely on simulated biofluids, controlled mechanical excitation, and short-term in vitro testing, with limited comparison against clinical reference methods or validation under long-term operating conditions [47]. Biosafety, wet-state stability, biofouling resistance, wearing comfort, and data standardization also require further investigation [48,49]. The field must therefore move beyond proof-of-signal demonstrations toward measurements that are reproducible, analytically selective, quantitatively reliable, and interpretable in clinically relevant contexts.

**Figure 1:**
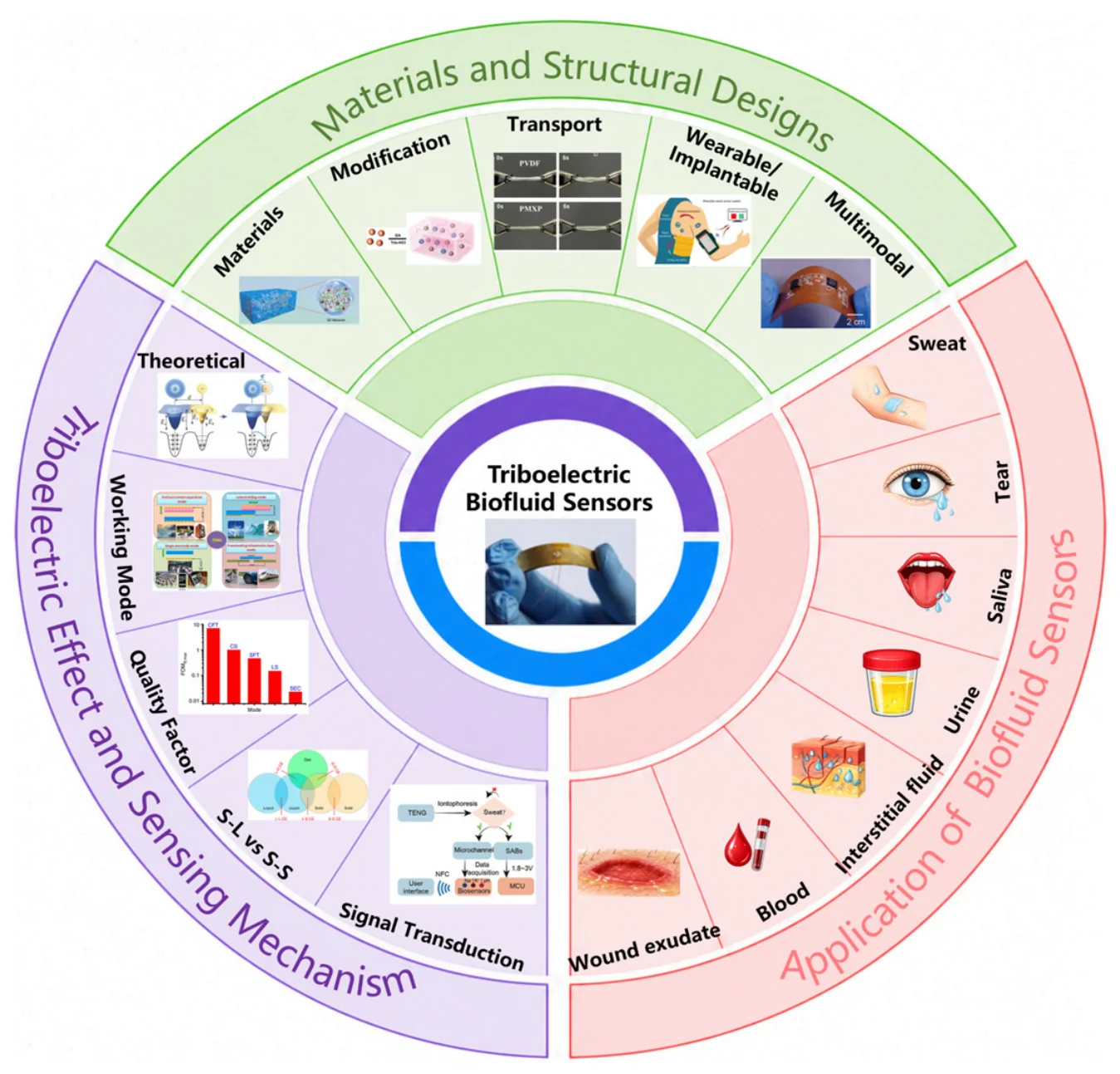
Overview of the theoretical foundations, material and structural designs, sensing configurations, and representative biofluid applications of BF-TENGs. Reprinted/adapted with permission from Ref. [50], Copyright 2012, Elsevier; Ref. [51], Copyright 2018, Wiley Online Library; Ref. [52], Copyright 2015, Elsevier; Ref. [53], Copyright 2015, Springer Nature; Ref. [54], Copyright 2022, American Chemical Society; Ref. [55], Copyright 2023, Wiley Online Library; Ref. [56], Copyright 2023, Elsevier; Ref. [57], Copyright 2023, Elsevier; [58] Copyright 2026, American Chemical Society; Ref. [59], Copyright 2020, Springer Nature.

This review summarizes recent progress in self-powered BF-TENG sensors for early disease screening, risk assessment, and auxiliary diagnosis (Figure 1). The theoretical foundation of triboelectric sensing is first discussed, including working modes, figures of merit, contact-charge transfer mechanisms, differences between solid–solid and solid–liquid triboelectrification, and biofluid signal transduction. Material selection, surface and interfacial functionalization, biofluid collection and transport, wearable and implantable configurations, and multimodal integration are then examined. Representative applications are organized according to biofluid source, covering sweat, ocular and tear-related systems, oral and saliva-related sensing, urine, interstitial fluid, blood, and wound exudate. Finally, the remaining challenges and future directions are analyzed in terms of early screening value, stability and accuracy, biocompatibility and biosafety, comfort and durability, biodegradation and sustainability, and standardization and clinical translation. By connecting fundamental interfacial mechanisms with device-level design and application-oriented validation, this review aims to guide the development of next-generation BF-TENG sensing systems.

## 2. Triboelectric Effect and Body Fluid Sensing Mechanism

### 2.1. Theoretical Foundation

**Figure 2:**
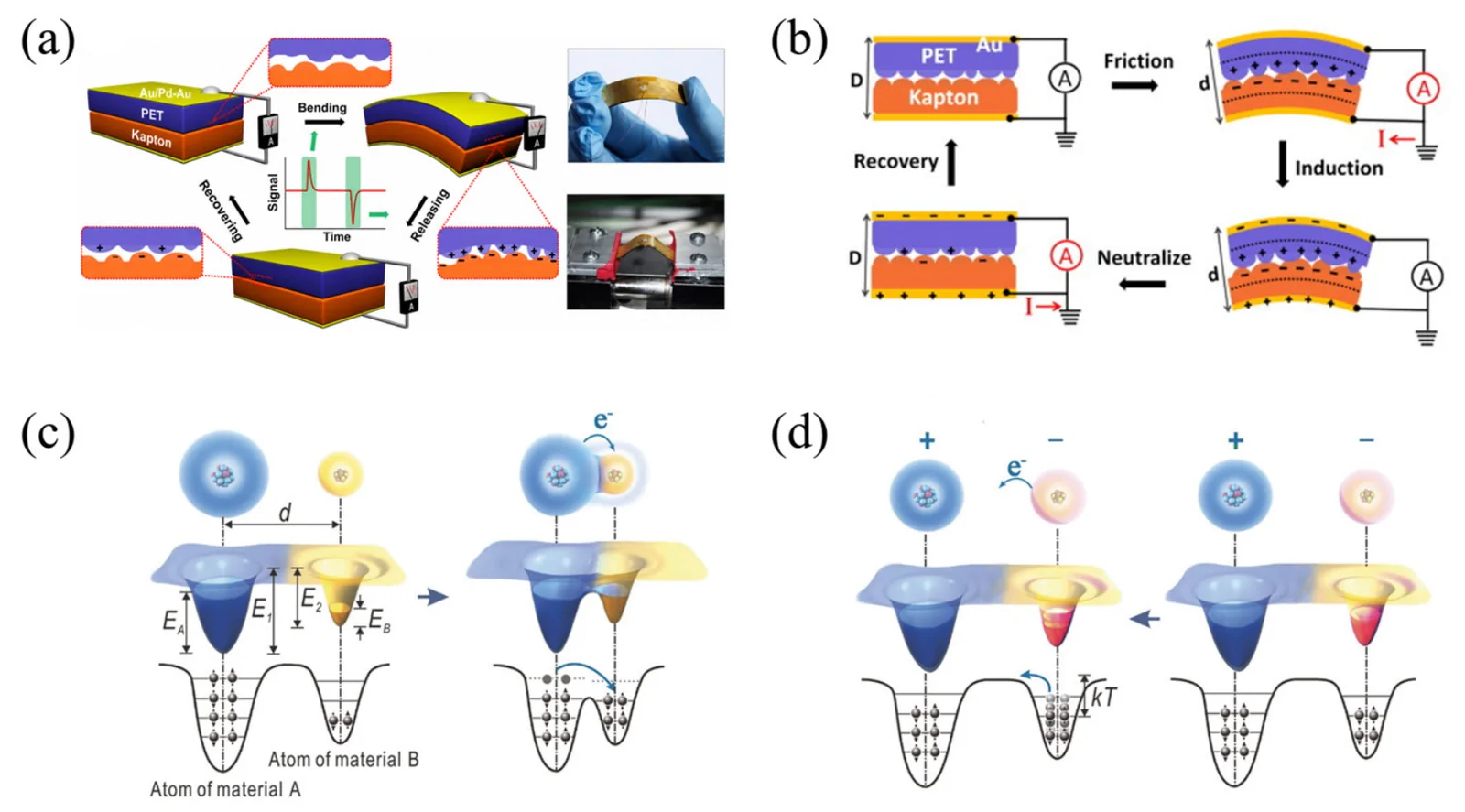
(**a**) The first TENG; (**b**) working mechanism of TENG. Reprinted/adapted with permission from Ref. [50], Copyright 2012, Elsevier. Electron-cloud potential-well model illustrating charge transfer before and after contact between materials without well-defined electronic band structures: (**c**) before contact and during contact; (**d**) after contact and electron release. Reprinted/adapted with permission from Ref. [51], Copyright 2018, Wiley Online Library.

Triboelectric nanogenerators (TENGs) were first proposed in 2012 (Figure 2a) [50]. Their operation is fundamentally based on the coupling between triboelectrification and electrostatic induction (Figure 2b). Among these processes, contact electrification plays a central role in charge generation, and its microscopic origin arises from a complex interplay of multiple interfacial phenomena, in which electron transfer is frequently considered a dominant contributor between contacting materials. This process is commonly interpreted using the electron-cloud potential-well model [51], as illustrated in Figure 2c,d. In this model, the electron cloud refers to the spatial distribution of outer electrons associated with specific atomic or molecular orbitals, while the corresponding atoms or molecules can be simplified as potential wells. Because outer-shell electrons are relatively weakly bound, they are more likely to participate in interfacial charge transfer during contact.

Before two materials come into contact, electron migration is restricted by the local potential wells of each material. Once material A contacts material B, their electron clouds overlap, and the original isolated potential wells evolve into an asymmetric double-well structure. This asymmetric potential landscape lowers the barrier for electron transfer in one direction, allowing electrons to move from material A to material B. Such a double-well configuration is conceptually analogous to the early-stage hydrogen bonding between OH and H groups [60], or to the potential-energy profile associated with proton transfer in NHN systems [61].

After separation, the transferred electrons can remain trapped on the surface of material B, particularly under relatively low-temperature conditions. During subsequent contact, the presence of an energy barrier prevents these electrons from spontaneously returning to their original sites. Only when sufficient thermal excitation is available can the trapped electrons migrate back to material A or dissipate into the surrounding environment through other pathways. It should be noted that electron transfer can occur between almost all contacting materials because interfacial potential differences are widely present. Nevertheless, contact electrification is rarely governed solely by electron transfer. Instead, it represents a coupled metaphysical process jointly mediated by electron transfer, ion migration, surface chemistry-driven species exchange, and interfacial polarization, whose relative contributions vary with material type, surface functional groups, humidity, and interfacial phase state. Although electron transfer is regarded as the dominant mechanism of contact electrification, other processes, such as ion migration and material transfer, may also contribute under specific conditions.

It is also important to recognize that TENG operation is closely associated with the polarization of dielectric materials. Its physical basis can be traced to the displacement current in the classical Maxwell equations. To account for the contribution of contact-electrification-induced electrostatic charges within Maxwell’s framework, Wang introduced an additional polarization term, $P_{s}$, into the electric displacement vector D in 2017 [62], which is defined as follows:

(1) $$D=\varepsilon_{0}E+P+P_{S}$$

Here, E denotes the electric field, P is the polarization induced by an external electric field, $P_{s}$ represents the polarization associated with surface charges generated by triboelectrification, and $\varepsilon_{0}$ is the vacuum permittivity. By substituting Equation (1) into Maxwell’s equations and defining $D^{\prime }=\varepsilon_{0}E+P$, the modified Maxwell equations can be expressed as follows:

(2) $$\nabla \cdot D^{\prime }=\rho_{f}-\nabla \cdot P_{S}$$

(3) ∇⋅B=0

(4) $$\nabla \times E=-\frac{\partial B}{\partial t}$$

(5) $$\nabla \times H=J_{f}+\frac{\partial D}{\partial t}$$

Here, ∇ denotes the Nable operator, B is the magnetic flux density, H is the magnetic field strength, $J_{f}$ is the free-current density, and $\rho_{f}$ is the free-charge density.

In Equation (6), the second term represents Maxwell’s displacement current, which is defined as follows:

(6) $$J_{D}=\frac{\partial D}{\partial t}=\varepsilon_{0}\frac{\partial E}{\partial t}+\frac{\partial P}{\partial t}$$

Displacement current was first introduced by Maxwell in 1861 to ensure consistency between Ampère’s law and the charge continuity equation [63]. The first term, $\frac{\partial E}{\partial t}$, describes the displacement current generated by a time-varying electric field and led to the development of electromagnetic-wave theory, which has transformed wireless communication, radar systems, antenna design, broadcasting, microwave transmission, telegraphy, and aerospace engineering. The second term, $\frac{\partial P_{S}}{\partial t}$, also known as the Wang term, represents the displacement current induced by an external strain field rather than by an electric field and constitutes the theoretical foundation of TENGs [64]. More broadly, the application of Maxwell’s equations to mechanically driven systems extend well beyond TENGs.

**Figure 3:**
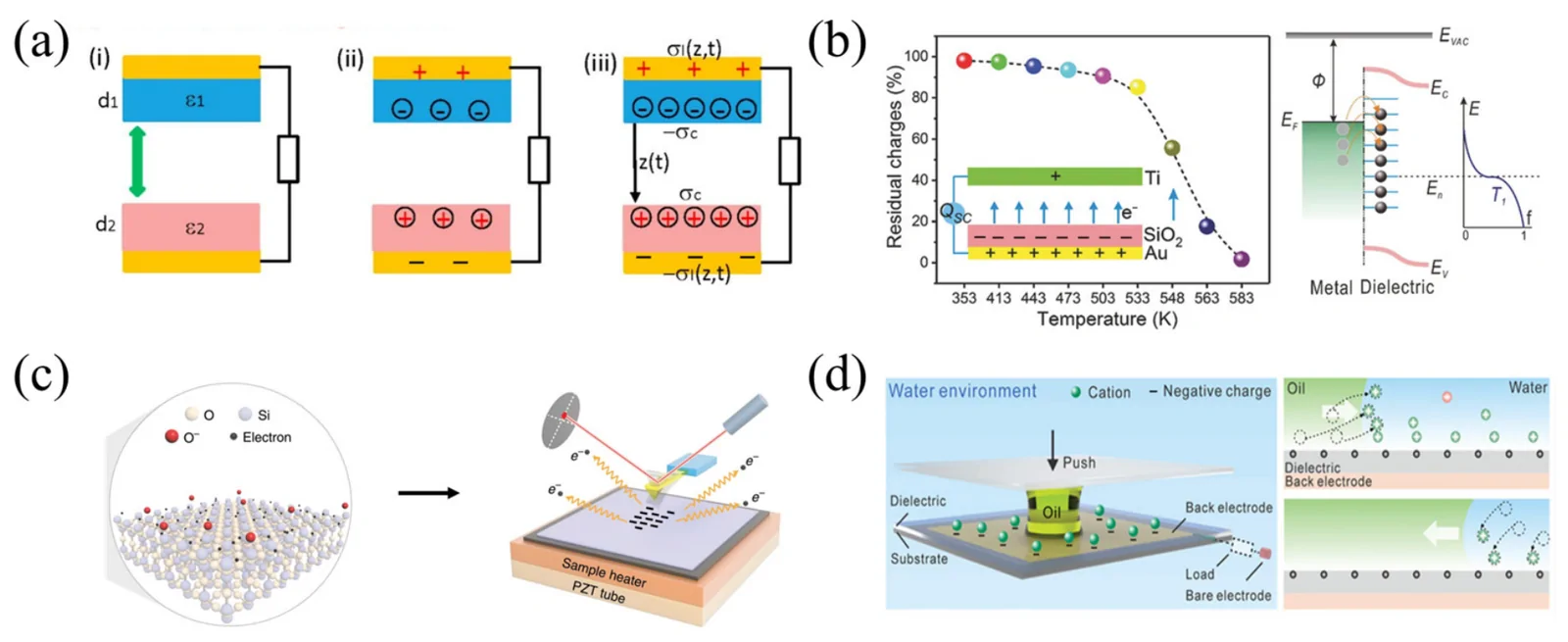
(**a**) Theoretical model of contact–separation type TENG: (i) initial configuration with two dielectric layers (ε_1_, ε_2_) and electrodes; (ii) triboelectric charge distribution after contact electrification; (iii) charge redistribution and induced current flow during the separation process, Reprinted/adapted with permission from Ref. [62], Copyright 2017, Elsevier. (**b**) Percentage of residual Q_SC_ of TENG after 5 min thermal treatment at different temperatures, inset shows the working mechanism diagram. Reprinted/adapted with permission from Ref. [51], Copyright 2018, Wiley Online Library. (**c**) Experimental setup for charge injection test. The negative charges accumulated on silica surface are probably derived from electrons and oxygen ions dissociated via surface ionization reaction (Si: silicon atom; O: oxygen atom; O^−^: oxygen anion). Reprinted/adapted with permission from Ref. [65], Copyright 2020, Springer Nature. (**d**) Schematic diagram of the TENG structure, operating mechanism, and oil diffusion and shrinkage behavior at the interface. Reprinted/adapted with permission from Ref. [66], Copyright 2022, Wiley Online Library.

As illustrated in Figure 3a, the operating mechanism can be explained using a contact–separation mode TENG. The device consists of two dissimilar dielectric materials, denoted as dielectric 1 and dielectric 2, with permittivity of $\varepsilon_{1}$ and $\varepsilon_{2}$ and thicknesses of $d_{1}$ and $d_{2}$, respectively. Under an external mechanical force, the two surfaces come into contact, and contact electrification induces charge transfer across the interface, leaving fixed charges of opposite polarity on the dielectric surfaces. These charges cannot move freely within the dielectric layers. Their surface charge density, $\sigma_{c}$, approaches saturation after repeated contact and remains independent of the separation distance z. During separation, the electrostatic field generated by these surface charges drives free electrons through the external circuit, producing a potential difference between the electrodes and thereby converting mechanical energy into electrical energy.

Another central issue in TENG research concerns the origin of transferred charges. Early studies predominantly adopted electron-transfer models, in which differences in metal work functions were used to explain the charging tendencies of dissimilar materials [67]. Whitesides and co-workers subsequently proposed an ion-transfer mechanism for solid–liquid contact [68], while other studies suggested that interfacial material transfer may also contribute to contact electrification [69]. Since 2018, Wang and co-workers have systematically investigated these mechanisms. For solid–solid contact, the observed charge decay followed thermionic-emission behavior, indicating that electron transfer generally dominates at both macroscopic and microscopic scales [51] (Figure 3b). At water–solid interfaces, electron and ion transfer may occur concurrently, with their relative contributions strongly influenced by surface wettability [65] (Figure 3c). Charge transfer at liquid–liquid [66,70] (Figure 3d), gas–solid [71], and other interfaces has also been extensively investigated.

As environmental humidity increases or a liquid phase participates in interfacial contact, the contribution of ionic processes generally becomes more pronounced. The formation of adsorbed water layers, dissociation of surface functional groups, and adsorption of solvated ions can collectively promote interfacial ion migration. At solid–liquid interfaces, these processes involve electrical double-layer formation and dynamic reconstruction, ion adsorption and desorption, and flow-induced charge redistribution. Kelvin probe force microscopy measurements further revealed the important contributions of ion adsorption and electrical double-layer rearrangement to interfacial charge generation, distinguishing solid–liquid electrification from dry solid–solid contact dominated primarily by electron transfer [72].

Nevertheless, solid–liquid contact electrification cannot be fully explained by ion adsorption within the conventional electrical double-layer model. By combining surface electronic-state analysis with first-principles calculations, Sun et al. showed that water adsorption induces local electron-density redistribution and interfacial polarization, thereby facilitating charge generation and transfer across the interface [73]. Changes in water-molecule orientation, hydrogen-bond networks, and the structure of the interfacial liquid layer can further modulate the electrical double layer and surface potential. Solid–liquid contact electrification should be regarded as a coupled multifactorial process involving synergistic electron transfer, ion migration, and interfacial polarization. These intertwined mechanisms collectively determine the polarity, density, and stability of interfacial charges, ultimately governing the energy-conversion efficiency and sensing sensitivity of TENGs.

### 2.2. Working Mode

**Figure 4:**
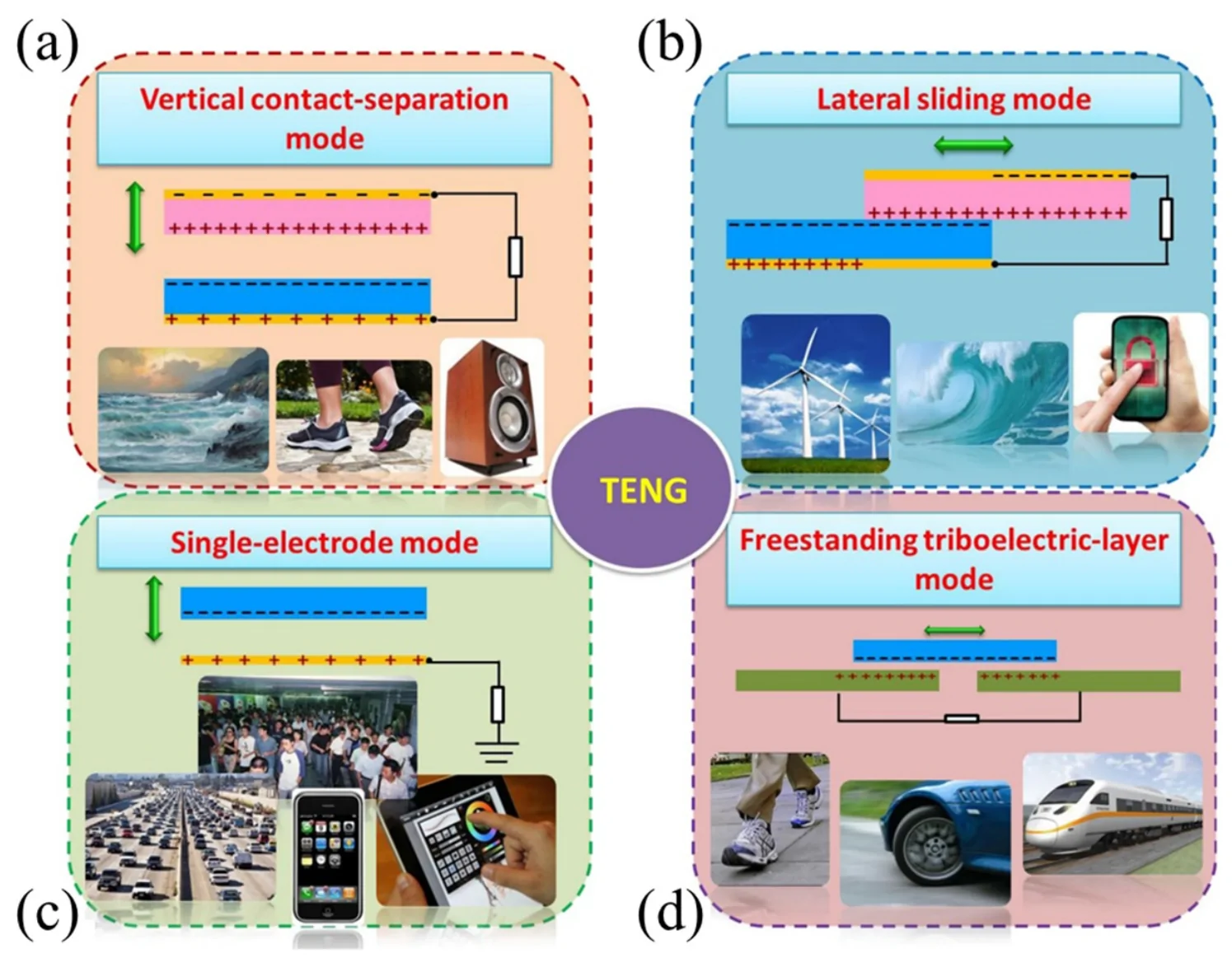
Four working configurations of triboelectric nanogenerators: (**a**) vertical contact-and-separation structure; (**b**) in-plane lateral sliding structure; (**c**) single-electrode induction structure; (**d**) movable free-standing triboelectric layer structure. Reprinted/adapted with permission from Ref. [52], Copyright 2015, Elsevier.

TENGs generally operate in four basic modes: vertical contact–separation, lateral sliding, single-electrode, and freestanding triboelectric-layer modes, as illustrated in Figure 4 [52]. Their selection depends on the form of mechanical input and device configuration. Contact–separation mode is commonly used for pressure or trigger sensing, whereas lateral sliding mode is more suitable for displacement detection. Single-electrode mode offers structural simplicity and freedom of motion but generally produces lower outputs and is more susceptible to environmental interference. Freestanding mode provides relatively simple fabrication and good device integration.

In the contact–separation mode, two triboelectric layers periodically contact and separate in the vertical direction. Contact electrification generates opposite surface charges, while separation creates a potential difference that drives electron flow through the external circuit. Repeated cycling produces an alternating electrical output.

The lateral sliding mode relies on parallel relative motion between two triboelectric layers. Changes in the overlapping contact area induce periodic variations in electrostatic potential, making this mode suitable for displacement and continuous motion sensing.

In the single-electrode mode, one triboelectric layer is connected to ground through an electrode, while the contacting object can move freely. Contact and separation alter the surface potential and drive electron flow between the electrode and ground. This simple and adaptable configuration is widely used in wearable and biofluid-related sensing.

The freestanding triboelectric-layer mode consists of a movable charged layer positioned above two fixed electrodes. Its motion changes the electrostatic potential distribution between the electrodes and generates an alternating current. This configuration is particularly suitable for integrated textiles and durable health-monitoring devices.

Each working mode offers distinct advantages and can be selected according to the mechanical input, device configuration, and application environment. Understanding these operating mechanisms provides the basis for designing TENGs for energy harvesting, self-powered sensing, and autonomous electronic systems.

### 2.3. Quality Factor

The four TENG working modes described above can be broadly classified into contact–separation TENGs (CS-TENGs) and sliding TENGs (S-TENGs). CS-TENGs include the conventional contact–separation mode (CS), single-electrode contact mode (SEC), and contact-mode freestanding triboelectric-layer mode (CFT), whereas S-TENGs comprise the lateral sliding mode (LS) and sliding freestanding triboelectric-layer mode (SFT). Although various combinations of materials and working modes have enabled diverse TENG configurations, a common metric can facilitate performance comparison when the underlying assumptions and testing conditions are clearly defined.

**Figure 5:**
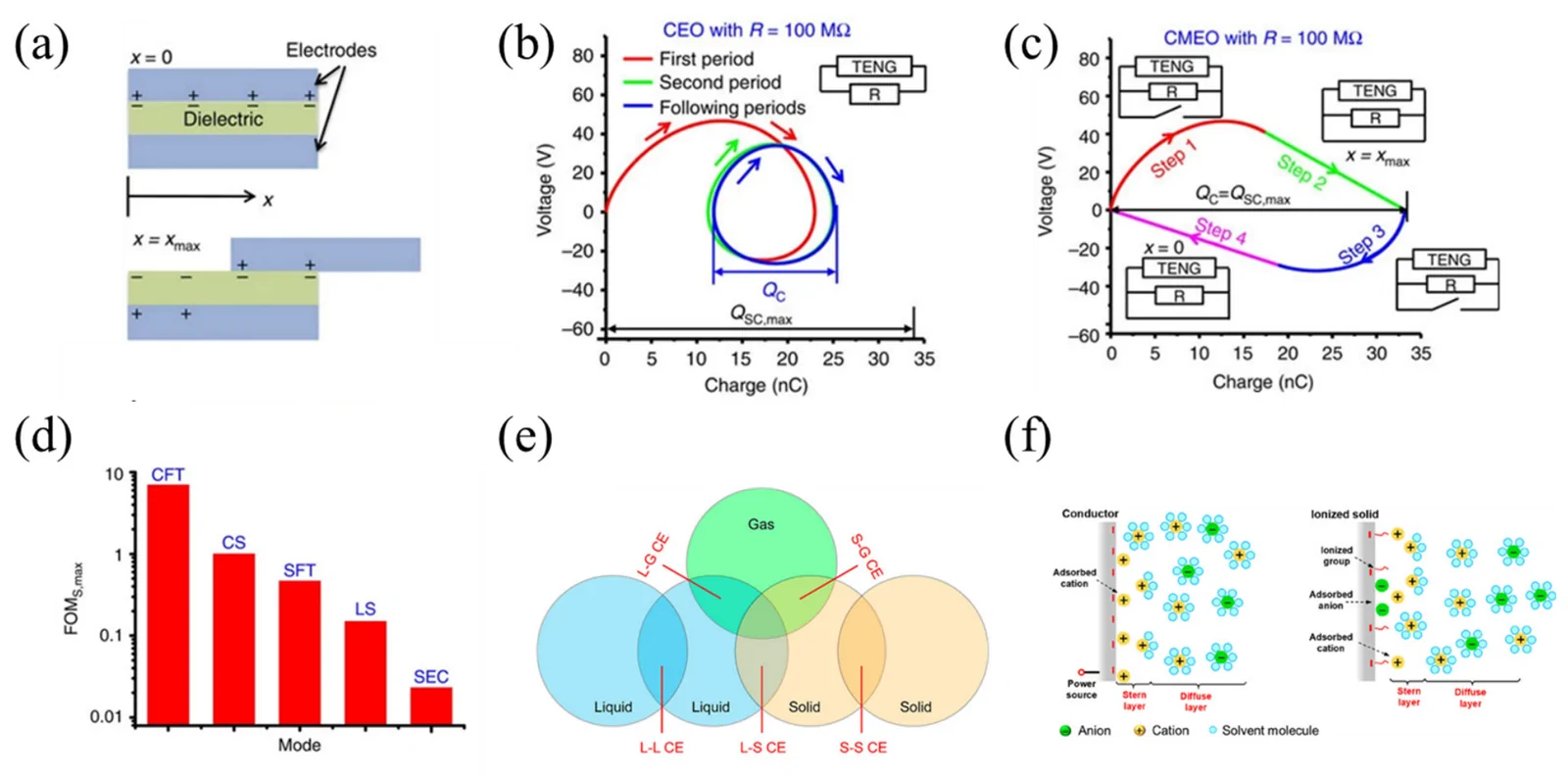
Merit and interfacial electrification mechanisms of TENGs: (**a**) Schematic of an LS-TENG; (**b**) CEO at 100 MΩ; (**c**) CMEO under the same condition; (**d**) maximum FOM_S_ for different operating modes. Reprinted/adapted with permission from Ref. [53], Copyright 2015, Springer Nature. (**e**) Contact electrification at solid–solid, liquid–solid, liquid–liquid, and gas-related interfaces; (**f**) conventional electrical double-layer models at electrode and charged solid surfaces, comprising compact and diffuse layers. Reprinted/adapted with permission from Ref. [54], Copyright 2022, American Chemical Society.

Zi et al. established a figure-of-merit (FOM) framework consisting of the structural FOM (FOM_s_), material FOM (FOM_m_), and performance FOM (FOM_p_) [53]. Taking an LS-TENG as an example (Figure 5a), finite-element simulations were used to establish the relationship between voltage V and transferred charge Q under different load resistances. The corresponding V–Q trajectory at a resistance of 100 MΩ is shown in Figure 5b. The enclosed area of the V–Q loop represents the electrical energy delivered during one operating cycle, referred to as the cycle for energy output (CEO). Once the TENG reaches a steady state, a closed V–Q loop is obtained, and its area varies with the external load resistance.

The maximum achievable loop area is defined as the cycle for maximized energy output (CMEO), providing a standardized basis for evaluating TENG performance (Figure 5c). Under the assumptions of the FOM model, the calculated structural FOM follows the order CFT > CS > SFT > LS > SEC (Figure 5d). This ranking is based on idealized geometries, charge distributions, prescribed mechanical motion, and optimized electrical loading. It represents the theoretical structural potential of each mode rather than a universal hierarchy, because experimental performance is also affected by material properties, effective contact, driving conditions, load matching, and energy losses. This framework separates the contributions of device structure and material properties, enabling more consistent comparison among TENGs with different configurations.

The structural figure of merit of FOM_S_ is defined as a parameter for evaluating device structural design. Here, $\varepsilon_{0}$ is the vacuum permittivity, and σ denotes the output surface charge density, expressed as the amount of charge per unit area in C/m^2^. The ratio $E_{m}/(Ax_{max})$ serves as a key indicator of TENG performance, where the maximum output energy $E_{m}$ is proportional to the square of the surface charge density, $\sigma^{2}$.

(7) $$FOM_{S}=\frac{2\varepsilon_{0}}{\sigma^{2}}=\frac{E_{m}}{Ax_{max}}$$

For the material figure of merit (*FOM_M_*), the surface charge density σ is the sole parameter used to evaluate triboelectric material performance. The establishment of the FOM framework provides a standardized benchmark for comparing TENG performance and may also support the industrial development of TENG-based systems. The performance figure of merit (*FOM_p_*), which directly reflects the maximum achievable average output power and energy-conversion efficiency of a TENG, can be expressed as follows:

(8) $$FOM_{p}=FOM_{s}\times \sigma^{2}=2\varepsilon_{0}\frac{E_{m}}{Ax_{max}}$$

The above equations enable the theoretical evaluation and comparison of different TENG configurations. Output performance can be improved by optimizing the structural figure of merit (*FOM_s_*) and increasing the maximum surface charge density, although the resulting FOM should be interpreted together with the actual operating conditions and loss mechanisms.

### 2.4. Solid–Liquid vs. Solid–Solid

Solid–solid TENGs (S–S TENGs) rely on the periodic contact, separation, or sliding of two solid surfaces and have been widely used for pressure, motion, and vibration sensing. Their practical performance, however, can be limited by interfacial wear caused by repeated friction, together with charge dissipation induced by humidity and surface contamination [74]. Solid–liquid TENGs (L–S TENGs) provide an alternative configuration in which dynamic liquid–solid contact generates electrical output with less mechanical abrasion. Their direct response to droplet impact, liquid flow, and pressure variation makes them particularly suitable for operation in complex fluid environments [75,76]. Contact electrification at the solid–liquid interface generally begins with interfacial charge-generation processes governed by the coupled interplay of electron transfer, ion migration, surface chemistry interactions, and interfacial polarization. When water or another polar liquid contacts highly electronegative materials such as PTFE or FEP, the solid surface typically acquires negative charges, followed by the redistribution of counterions in the adjacent liquid phase [77,78]. As illustrated in Figure 5e,f, this interfacial organization can be described by the electrical double-layer model. The compact Stern layer contains relatively immobile counterions near the charged surface, whereas ions in the outer diffuse layer remain dynamically distributed according to the interfacial electric field [54]. Droplet spreading, sliding, or separation subsequently alters the electric-field distribution and induces electron flow through the external circuit, thereby producing an alternating electrical output [79].

The hybrid electrical double-layer model further indicates that electron transfer and ion redistribution occur sequentially rather than as mutually exclusive mechanisms. Upon liquid–solid contact, overlap of the interfacial electron clouds lowers the energy barrier for charge exchange, enabling initial electron transfer to the solid surface. Counterions in the liquid then adsorb and reorganize near the charged interface, completing the electrical double-layer structure [80]. Solid–liquid triboelectrification should therefore be understood as a multifactorial, coupled process arising from synergistic electron transfer, ion redistribution, surface chemical effects, and interfacial polarization, rather than being dominated by a single charge transfer pathway.

The main distinction between S–S and L–S systems lie in charge retention and environmental responsiveness. Charges generated at dry solid interfaces can be trapped in surface states and often exhibit relatively high densities and long retention times. By contrast, interfacial charges in liquid environments are continuously modulated by ion concentration, pH, solvent properties, adsorption, diffusion, and convection, leading to faster charge relaxation and dissipation [81]. Although L–S TENGs generally produce lower outputs than their solid–solid counterparts, their strong sensitivity to ionic composition and interfacial chemistry makes them particularly attractive for the analysis of sweat, saliva, urine, and other biofluids [82]. Accordingly, S–S TENGs are better suited to mechanical sensing and energy harvesting, whereas L–S TENGs provide a more direct route toward real-time, self-powered detection of chemical and physiological information.

### 2.5. Signal Transduction

TENG/TENSor platforms serve not only as mechanical energy harvesters but also as interfacial charge-to-signal transducers. Variations in the electrical double layer, surface charge, and contact state induced by biofluids can be converted into measurable open-circuit voltage, short-circuit current, or transferred charge.

Ionic strength modulates the effective charge at the solid–liquid interface by altering the electrical double-layer thickness and electrostatic screening. Increasing ion concentration generally compresses the electrical double layer and reduces the effective surface potential, although the resulting response also depends on ion valence, specific adsorption, and solution conductivity. Meanwhile, pH regulates the protonation and deprotonation of functional groups such as carboxyl, amino, and hydroxyl groups, thereby changing the interfacial ζ-potential, triboelectric polarity, and charge density. Consequently, pH can function either as an independent sensing target or as a correction parameter for other biomarker measurements.

Biofluid viscosity primarily affects droplet spreading, retraction, residence time, and separation dynamics, which are reflected in the amplitude and waveform of the triboelectric output. Biomolecules such as proteins, glucose, and lactate can additionally alter solution conductivity and dielectric properties or interact specifically with functional interfaces, leading to changes in surface charge, wettability, and interfacial potential. Recognition layers based on enzymes, molecularly imprinted polymers, or conducting polymers can therefore encode these chemical variations into concentration-dependent electrical signals under identical mechanical excitation. With appropriate calibration, circuit design, and signal processing, TENG/TENSor platforms can support real-time, self-powered, multiparameter analysis of sweat, saliva, urine, and other biofluids.

## 3. Materials and Structural Designs for Triboelectric Biofluid Sensors

The materials and structural design of triboelectric biofluid sensors are strongly dependent on the intended application scenario. Unlike conventional pressure or motion sensors, these devices operate in wet, saline, protein-rich, and contamination-prone environments. Their performance is therefore governed not only by triboelectric output, but also by fluid spreading, interfacial fouling, sample transport, mechanical conformity, and biosafety. Accordingly, material and structural design should extend beyond maximizing voltage output to support efficient biofluid collection, interfacial stability, reliable signal readout, and long-term operation.

### 3.1. Selection of Materials

Materials used in BF-TENGs can be broadly classified into high-charge-output materials, soft bio-interface materials, and functional composites. Fluorinated and polar polymers exhibit strong charge-trapping capability and good film-forming properties, making them suitable for droplet-driven devices and skin-contact triboelectric layers. However, salts, proteins, and other complex constituents in biofluids accelerate charge dissipation. Wet-state stability is therefore often more practically important than high electrical output alone.

Natural polymers, ionic hydrogels, and elastic conductive gels feature low moduli, high water content, and good conformability, making them particularly suitable for interfaces involving the skin, wounds, and interstitial fluid. Their value extends beyond biocompatibility to reducing mechanical mismatch between the device and biological tissue.

**Figure 6:**
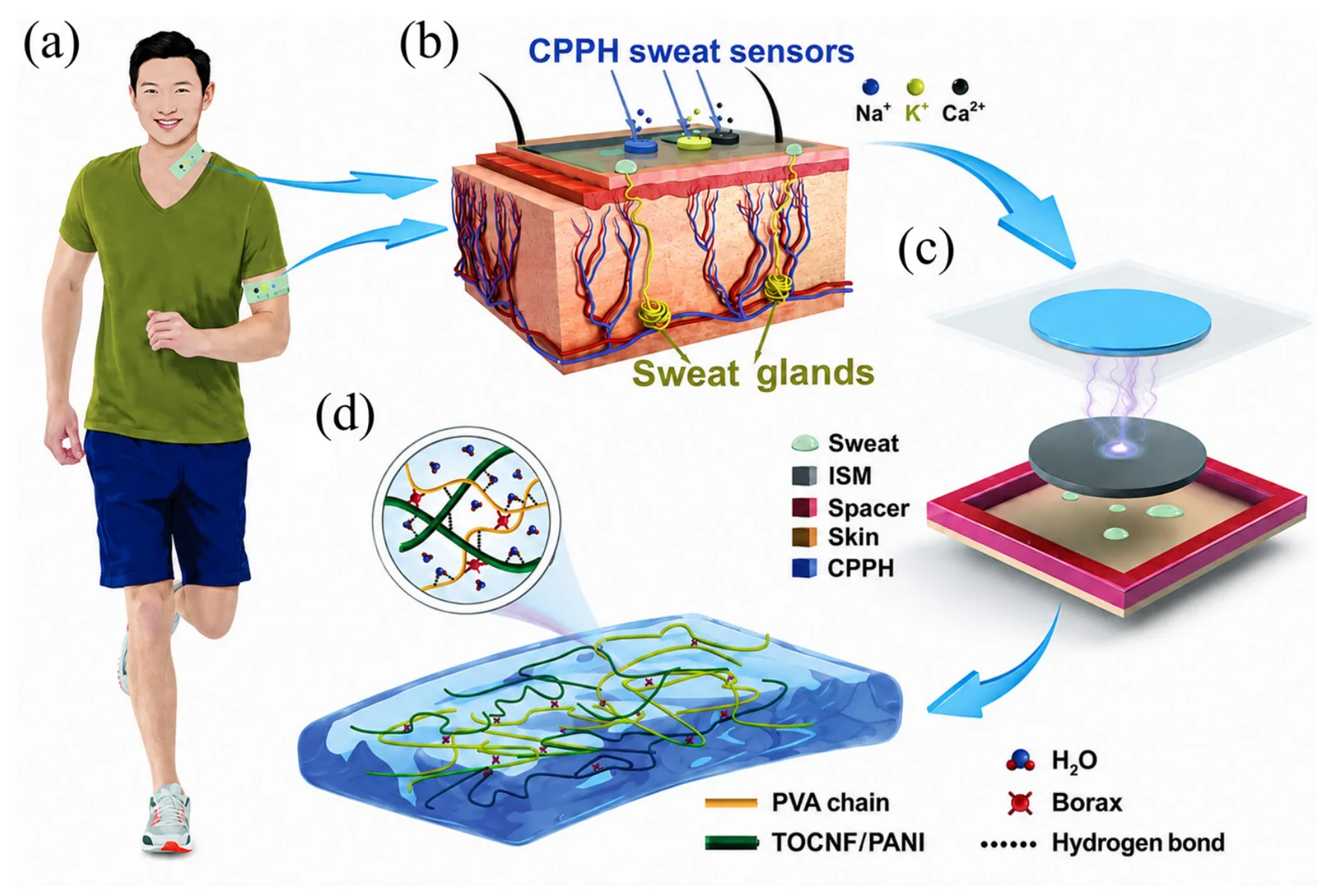
Cellulose-based hydrogel for self-powered sweat sensing. (**a**) On-body ion monitoring; (**b**) detection of Na^+^, K^+^, and Ca^2+^; (**c**) sensor configuration; (**d**) electrode structure and morphology. Reprinted/adapted with permission from Ref. [83], Copyright 2022, Wiley Online Library.

For example, Qin et al. constructed a stretchable and self-healing sweat sensor using a nanocellulose/polyaniline conductive hydrogel [83]. The device enabled real-time monitoring of sweat ions during body movement (Figure 6a). As illustrated in Figure 6b, the sensor was positioned over sweat glands to quantify Na^+^, K^+^, and Ca^2+^. Its layered sensing configuration is presented in Figure 6c, whereas the interconnected conductive network of the CPPH electrode is shown in Figure 6d. The hydrogel recovered over 95% of its mechanical and electrical performance within 10 s and exhibited a stretchability of 1530%, supporting stable and conformable on-body analysis.

Functional composites are primarily introduced to enhance charge collection, stabilize interfaces, or provide additional biological functions [84,85,86]. Although two-dimensional materials, dielectric fillers, and antimicrobial nanocomponents can improve electrical or antibacterial performance, they may also introduce aggregation, oxidation, and potential biosafety concerns [87,88]. Each component should therefore serve a clearly defined role rather than being incorporated through simple material stacking. Qu et al. modified a PDMS triboelectric layer with PTFE particles and combined it with a Ag nanowire/PVA hydrogel electrode to construct a flexible superhydrophobic TENG [89]. The low-surface energy composite interface reduced moisture-induced interference with charge transfer and retention, allowing the device to rapidly recover and maintain a relatively high output under humid conditions.

Overall, material selection for BF-TENGs should shift from a triboelectric-series-driven approach toward an application-environment-oriented strategy. Sweat monitoring requires breathability, flexibility, and resistance to salt interference, whereas wound and blood sensing place greater emphasis on antifouling and antibacterial performance. Interstitial-fluid monitoring and implantable applications should prioritize tissue compatibility, degradation safety, and operational lifetime. The key criterion is not the highest output, but the ability to maintain stable functionality in the intended biofluid environment.

### 3.2. Modification and Functionalization

Surface modification governs the interfacial response when biofluids contact the device. Real biofluids contain ions, proteins, and cellular debris that interact continuously with the triboelectric surface. Biofouling therefore evolves over time: initial ion adsorption and electrical double-layer reorganization are followed by protein adsorption and rearrangement, whereas prolonged exposure may lead to salt accumulation, cellular or bacterial adhesion, and biofilm formation. These progressive changes alter the surface charge density, wettability, effective contact area, and charge retention, leading to baseline drift and reduced sensitivity. Surface functionalization should therefore extend beyond enhancing charge generation to include fluid regulation, antifouling protection, and selective recognition.

**Figure 7:**
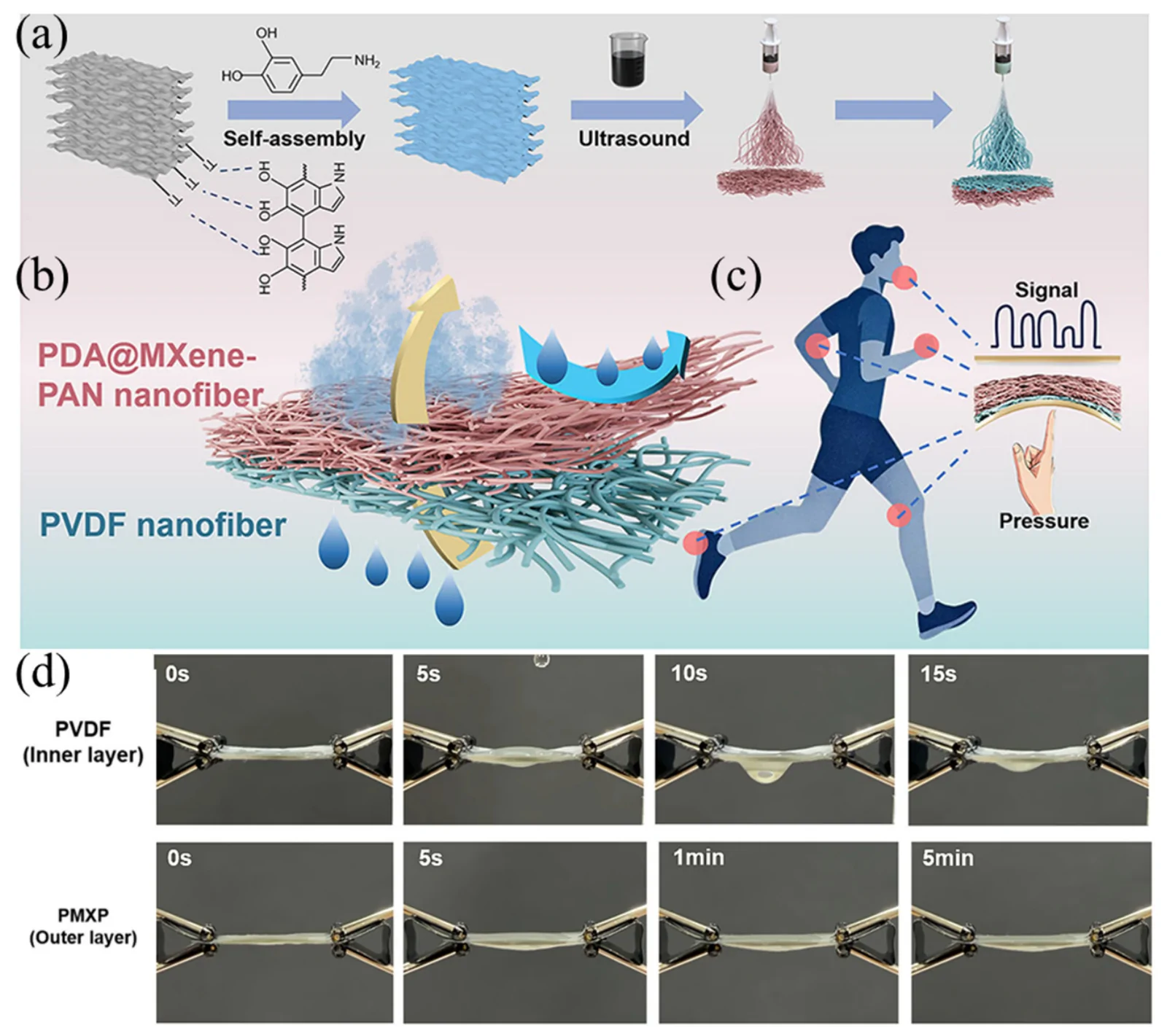
(**a**) Fabrication of the PDA@MXene–PAN/PVDF Janus nanofibrous membrane; (**b**) directional water transport through the Janus bilayer membrane; (**c**) self-powered monitoring of human motion and physiological signals; (**d**) schematic illustration of unidirectional water transport through the composite membrane. Reprinted/adapted with permission from Ref. [58]. Copyright 2026, American Chemical Society.

Wettability and micro/nanostructures jointly control biofluid spreading, transport, and removal. Hydrophilic interfaces facilitate the rapid entry of trace samples into sensing regions, whereas hydrophobic surfaces favor droplet contact–separation and self-cleaning. Janus interfaces and wettability gradients can further enable directional transport. For example, Mei et al. constructed a bilayer Janus membrane comprising a hydrophobic PVDF layer and a hydrophilic PDA@MXene–PAN layer, as illustrated in Figure 7a [58]. Gradients in pore size and wettability promoted directional water transport across the membrane, while the PDA@MXene network provided abundant charge-trapping sites (Figure 7b). The resulting TENG generated an open-circuit voltage of approximately 150 V and a peak power density of 302.6 mW m^−2^, enabling self-powered monitoring of gait, respiration, and joint motion (Figure 7c). Unidirectional water transport was experimentally achieved within 10 s, whereas reverse permeation remained strongly suppressed (Figure 7d). This design effectively balanced moisture management and triboelectric performance. Nevertheless, excessive surface roughness may promote protein adsorption, salt deposition, and bacterial adhesion, requiring careful coordination of charge generation, fluid removal, and interfacial cleanliness.

Long-term sensing reliability and specificity also depend on the coordination of antifouling and biorecognition layers. Antifouling polymers, polydopamine interlayers, and antimicrobial components can mitigate interfacial passivation, whereas ion-selective membranes, enzymes, antibodies, aptamers, and molecularly imprinted layers provide target-specific recognition. In a complementary approach, Song et al. incorporated CeO_2_ nanorods and MXene into an amyloid albumin hydrogel and coated the composite onto flexible screen-printed electrodes [90]. The hydrated hydrogel layer suppressed nonspecific adsorption from sweat, while CeO_2_ imparted antimicrobial activity, thereby improving the stability of dopamine detection in human sweat. An effective functional interface should ultimately preserve recognition activity and resist biofouling while maintaining stable electrical readout.

### 3.3. Biofluid Collection and Transport

Biofluid collection and transport structures determine whether TENGs can obtain stable and analytically meaningful samples. Real biofluids are often limited in volume, irregularly produced, and susceptible to evaporation, contamination, and mechanical disturbance. Without reliable fluid management, electrical outputs are difficult to correlate accurately with physiological changes.

**Figure 8:**
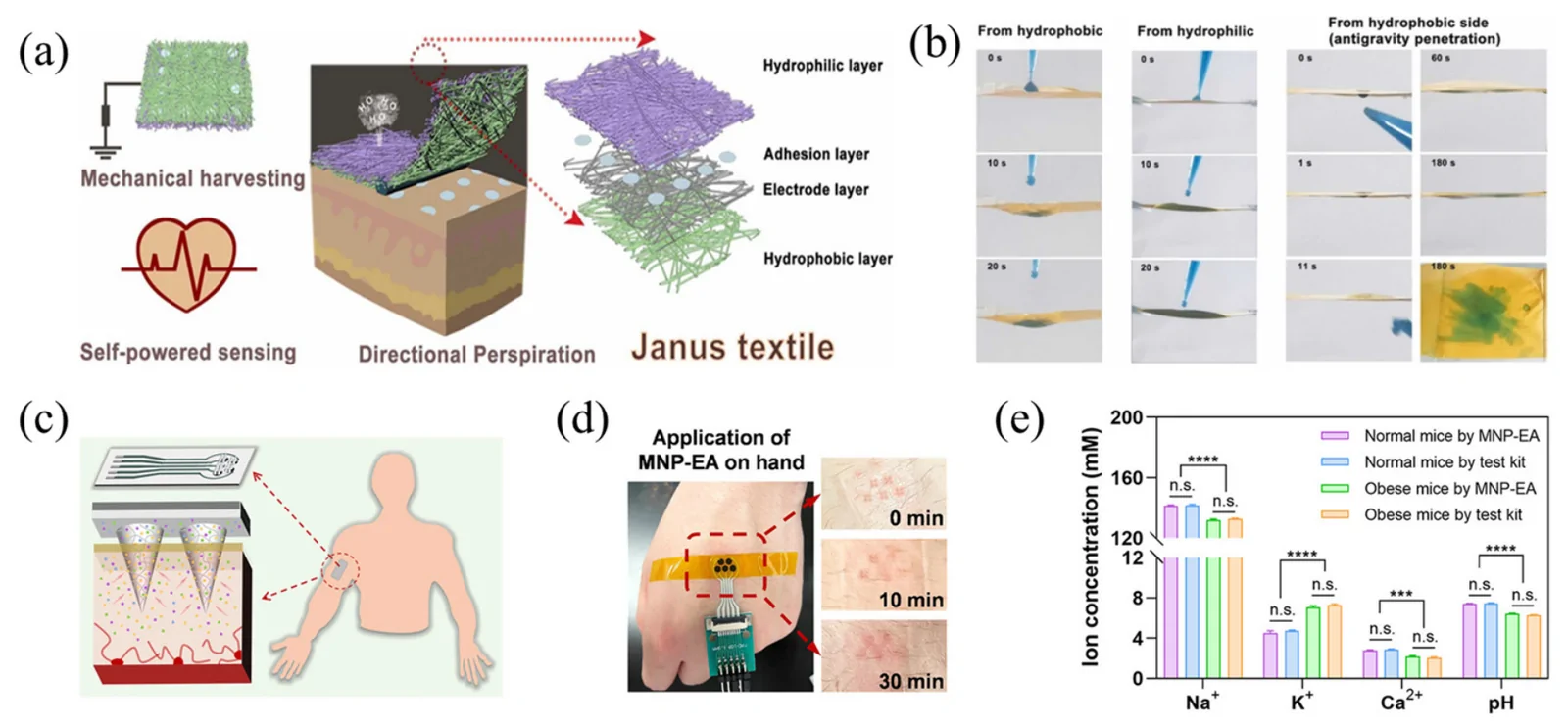
(**a**) Structural features and practical applications of Janus fabrics; (**b**) optical photographs of unidirectional water transportation process. Reprinted/adapted with permission from Ref. [91]. Copyright 2023, Elsevier. (**c**) Preparation strategy and application scenarios of the MNP-EA composite device; (**d**) optical images showing the MNP-EA device attached to the human hand for wearable testing; (**e**) comparison of ion concentrations in normal mice and obese mice detected by the MNP-EA sensor and commercial detection kits, *** *p* < 0.001; **** *p* < 0.0001; n.s. = not significant, Reprinted/adapted with permission from Ref. [92]. Copyright 2023, American Chemical Society.

Flexible porous materials, including fibrous membranes, textiles, and hydrogels, enable pump-free transport through capillary action while maintaining breathability and conformability. Janus interfaces and wettability gradients can further guide biofluids toward sensing regions or facilitate rapid removal, making them particularly suitable for sweat and wound exudate management. Their pore structure and fluid-retention capacity, however, must be carefully controlled to avoid fluid accumulation, delayed responses, and local overhydration. For example, Cheng et al. developed an all-nanofibrous Janus textile integrating directional perspiration transport, mechanical energy harvesting, and self-powered sensing (Figure 8a) [91].The textile comprised a hydrophilic small-pore HPAN/BNNS layer, a conductive Ag nanowire layer, and a hydrophobic large-pore TPU layer. Capillary pressure and the wettability gradient promoted rapid water transport from the hydrophobic side to the hydrophilic side while suppressing reverse penetration, even against gravity (Figure 8b). When used as a TENG-based electronic skin, the textile generated an open-circuit voltage of 78.10 V and retained a detectable output under wet conditions, indicating that directional fluid management can improve the reliability of wearable physiological sensing.

Interstitial fluid collection typically requires microneedle-based structures. Microneedle arrays can penetrate the stratum corneum to access interstitial fluid, whose composition is closely correlated with that of blood, while reducing the invasiveness associated with conventional blood sampling. For example, Zhu et al. developed a flexible microneedle patch–electrode array (MNP-EA) that integrated interstitial fluid extraction with multiplexed ion detection (Figure 8c) [92]. Swellable microneedles collected interstitial fluid and delivered it to ion-selective electrodes for the simultaneous analysis of Na^+^, K^+^, Ca^2+^, and H^+^. The patch was further evaluated on the human hand (Figure 8d), and measurements obtained from normal and obese mice showed good agreement with commercial test kits (Figure 8e).

### 3.4. Wearable and Implantable Configurations

Biofluid source determines not only the sampling site but also the mechanical loading mode and interfacial architecture of the device. Sweat sensing is commonly implemented using skin patches or textiles, whereas interstitial fluid sampling typically relies on microneedles to access superficial tissue layers, and wound exudate monitoring is more readily integrated with dressings. Accordingly, the design of wearable and implantable BF-TENGs should focus not merely on device miniaturization, but on matching the sampling pathway, triboelectric interface, and mechanical stimulus to the specific biological interface.

**Figure 9:**
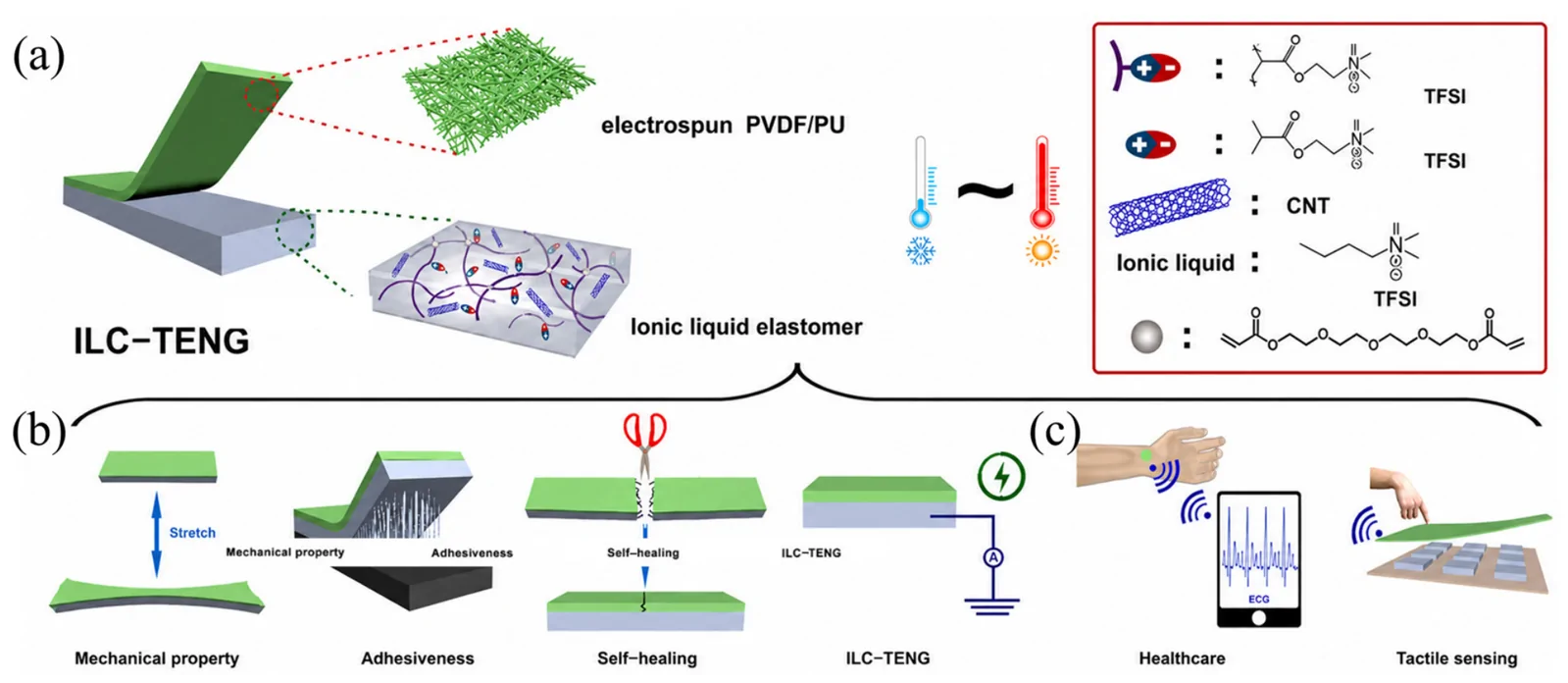
(**a**) Structural design and material composition of the ILC-TENG; (**b**) mechanical, adhesive, self-healing, and energy-harvesting properties; (**c**) self-powered healthcare monitoring and tactile sensing applications. Reprinted/adapted with permission from Ref. [93]. Copyright 2022, Elsevier.

Skin patches represent the most widely used wearable configuration, yet their performance depends strongly on maintaining a stable interface under motion and humid conditions. Sweat can alter surface charge and frictional behavior, while skin deformation may induce partial delamination, making it difficult to distinguish physiological variations from device drift. Patch design must therefore balance mechanical compliance, breathability, and adhesive stability. Notably, Cheng et al. developed an autonomously adhesive triboelectric skin based on an ionic liquid elastomer (Figure 9a) [93]. A CNT-doped ionic liquid elastomer served as the flexible electrode, while an electrospun PVDF/PU nanofibrous membrane functioned as the triboelectric layer. The device combined stretchability, self-adhesion, self-healing, and energy harvesting (Figure 9b), with recoverable output after 100% stretching, mechanical damage, and washing. As shown in Figure 9c, it further enabled joint-motion and electrocardiographic monitoring, as well as tactile sensing, highlighting the importance of robust adhesion and deformation tolerance in wearable TENGs.

Implantable BF-TENGs shift the primary concern from interfacial stability to in vivo safety and long-term functionality. These devices must withstand continuous tissue motion while minimizing signal attenuation caused by mechanical mismatch, biofluid ingress, and fibrotic encapsulation. In biodegradable systems, the degradation rate must be aligned with the intended monitoring period, whereas nondegradable devices require careful consideration of encapsulation failure and eventual surgical removal. For example, Zheng et al. developed a lifetime-tailorable biodegradable TENG for harvesting biomechanical energy in vivo [94]. Its electrical output and degradation period could be adjusted by varying the constituent materials, allowing the device to degrade and be resorbed after completing its intended operation. The generated energy was further used to power micrografting electrodes and produce pulsed electric fields that guided directional nerve cell growth.

### 3.5. Multimodal Design

BF-TENGs for early disease screening usually require multimodal integration rather than reliance on a single triboelectric unit. Biofluid sensing involves several sequential processes, including sample collection, transport, recognition, and signal readout. Therefore, TENGs are often integrated with microfluidics, electrochemical sensors, chemical recognition interfaces, visual readout modules, flexible circuits, or therapeutic units. The purpose is not to simply stack multiple functions, but to improve the completeness and reliability of detection through rational functional division.

In different systems, TENGs can serve either as energy units or sensing units. As energy units, they harvest energy from human motion, droplet flow, or interfacial contact to power low-consumption sensing modules. As sensing units, they respond to biofluid flow, pressure variation, wetting state, or interfacial contact behavior. Clarifying the functional role of TENGs helps avoid mistaking high electrical output for high detection accuracy.

**Figure 10:**
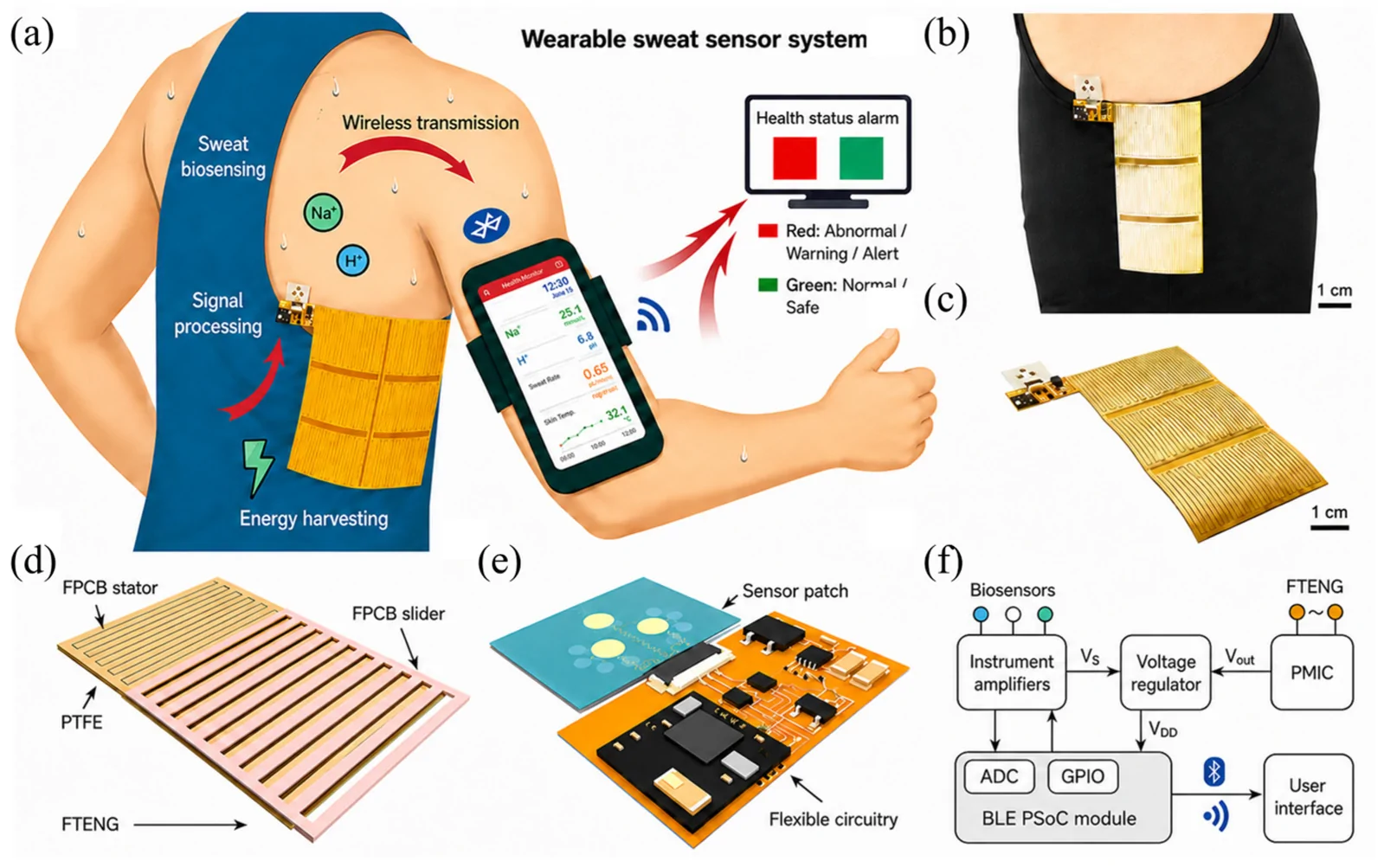
FTENG-powered wearable sweat sensor system (FWS3) device for wireless non-invasive biomolecule detection: (**a**) Schematic of FWS3 integrating body motion energy collection, signal conditioning, microfluidic sweat sensing and Bluetooth wireless communication for real-time health monitoring on mobile devices. (**b**,**c**) Photographs of FPCB-supported FWS3 worn on human lateral torso. Scale bars, 4 cm. (**d**) Structure schematic of FPCB-based FTENG composed of grating slider and interdigital stator. (**e**) Layout of FWS3 with microfluidic sweat sensing patch connected to flexible circuits. (**f**) System block diagram describing power management, signal conversion, data processing and wireless transmission from FTENG and biosensors to user terminals. Reprinted/adapted with permission from Ref. [59]. Copyright 2020, Springer Nature.

Electrochemical modules are among the most widely adopted components in BF-TENG systems because they complement the energy-harvesting capability of TENGs with molecular selectivity and quantitative detection. This functional division allows TENGs to provide self-powered operation or auxiliary signal acquisition, while electrochemical sensors perform the specific analysis of low-abundance biomarkers in complex biofluids. Song et al. reported a wireless, battery-free sweat-sensing system powered by body motion [59]. The overall platform combined biomechanical energy harvesting, sweat analysis, signal processing, and Bluetooth communication for real-time health monitoring (Figure 10a). The flexible device could be worn on the lateral torso, as shown in Figure 10b,c. Its freestanding TENG consisted of a grating slider and an interdigitated stator supported by a flexible printed circuit board (Figure 10d), achieving a power density of approximately 416 mW m^−2^. The TENG, microfluidic sensor patch, and flexible electronics were integrated into a compact wearable configuration (Figure 10e). Power management, signal conversion, data processing, and wireless transmission from the biosensors to the user interface are summarized in Figure 10f. This system illustrates the coordinated integration of energy harvesting, biochemical sensing, and wireless communication for self-sustained biofluid monitoring. However, turning the individual components into a fully functional system remains difficult. The pulsed output of TENG must be converted into a stable supply, which introduces rectification losses and cold-start requirements. Supercapacitors and micro-batteries add self-discharge and cycle-life constraints, while wireless transmitters require high instantaneous power. Peak TENG output alone does not describe this energy chain; end-to-end efficiency, charging time, and achievable duty cycle are more informative at the system level.

### 3.6. Mechanical–Chemical Signal Decoupling

Although multimodal integration improves the functional completeness of BF-TENG systems, it also complicates signal interpretation. Triboelectric output is jointly affected by interfacial chemistry and mechanical excitation [95]. Consequently, changes in force, motion frequency, contact area, liquid volume, or flow rate may be incorrectly attributed to variations in biomarker concentration, particularly when the triboelectric interface is used directly for chemical sensing.

Differential sensing and reference channels provide practical approaches to reducing such interference [96]. The sensing and reference channels should have similar geometries, surface properties, and mechanical responses, while only the sensing channel contains the specific recognition element. Differences or ratios between their outputs can reduce common disturbances caused by motion and fluid flow. Microfluidic components, such as metering chambers, capillary valves, and flow resistors, can further regulate sample volume, flow rate, and contact area.

Mechanical parameters may also be recorded using pressure, strain, or flow sensors and incorporated into baseline correction, normalization, or multivariable analysis [97]. To verify effective decoupling, biomarker concentration and mechanical conditions should be varied independently. The corrected output should remain sensitive to the target analyte while showing limited dependence on force, frequency, or flow rate. Combining reference channels, controlled fluid delivery, and signal correction is therefore important for reliable BF-TENG-based biofluid analysis.

## 4. Application of Triboelectric Biofluid Sensors in Early Disease Diagnosis

Biofluid sensing represents an important entry point for the transition of triboelectric nanogenerators from energy harvesting toward biomedical diagnosis. Unlike conventional analytical methods that rely on external power supplies and rigid detection instruments, triboelectric biofluid sensors typically generate electrical signals from human motion, droplet contact, liquid flow, or interfacial wetting processes, thereby enabling self-powered, flexible, and continuous monitoring to some extent. However, TENGs play distinct technological roles in these systems: they may serve as direct sensing interfaces modulated by biofluid composition or interfacial behavior, as energy sources for electrochemical, wireless, iontophoretic, or therapeutic modules, or as biomechanical sensors of biofluid-related physiological activities. Because different biofluids vary substantially in sampling mode, biomarker concentration, contamination risk, and clinical interpretability, each application requires tailored interfacial materials, device architectures, and signal transduction strategies.

### 4.1. Sweat-Based TENSor

Sweat is currently one of the most extensively studied biofluids in wearable sensing, primarily because it can be collected noninvasively, is continuously available under appropriate physiological conditions, and is naturally compatible with flexible electronics on the skin surface. Sweat contains electrolytes such as Na^+^, K^+^, and Cl^−^, as well as metabolism-related molecules including lactate, urea, glucose, amino acids, and cortisol. These constituents can be used to assess dehydration, electrolyte imbalance, exercise metabolism, muscle fatigue, and stress status. Compared with blood analysis, the main advantage of sweat sensing does not lie in the absolute accuracy of a single measurement, but in its suitability for long-term, dynamic, and context-specific monitoring.

In sweat-based TENSor systems, triboelectric signals generally originate from two types of processes. In the first, human motion drives solid–solid contact–separation or sliding friction, where the TENG mainly serves as an energy unit to power sweat sensing modules. In the second, sweat droplets or continuous liquid flow contact functional surfaces to generate solid–liquid triboelectric signals, allowing the TENG itself to function as a sensing unit. The former configuration is more suitable for integration with electrochemical or ion-selective electrodes for quantitative analysis of specific ions or metabolites, whereas the latter is more dependent on fluid flow rate, ionic strength, surface charge density, and wetting state, making it suitable for constructing physical sensing models of sweat secretion rate or sweat transport behavior.

In recent years, microfluidic architectures have markedly improved the reliability of sweat-based TENSor. By incorporating capillary-driven channels, compartmentalized reservoirs, and directed transport pathways, sweat can be guided to predefined sensing regions, thereby minimizing signal drift caused by uncontrolled spreading, evaporation, and contamination.

**Figure 11:**
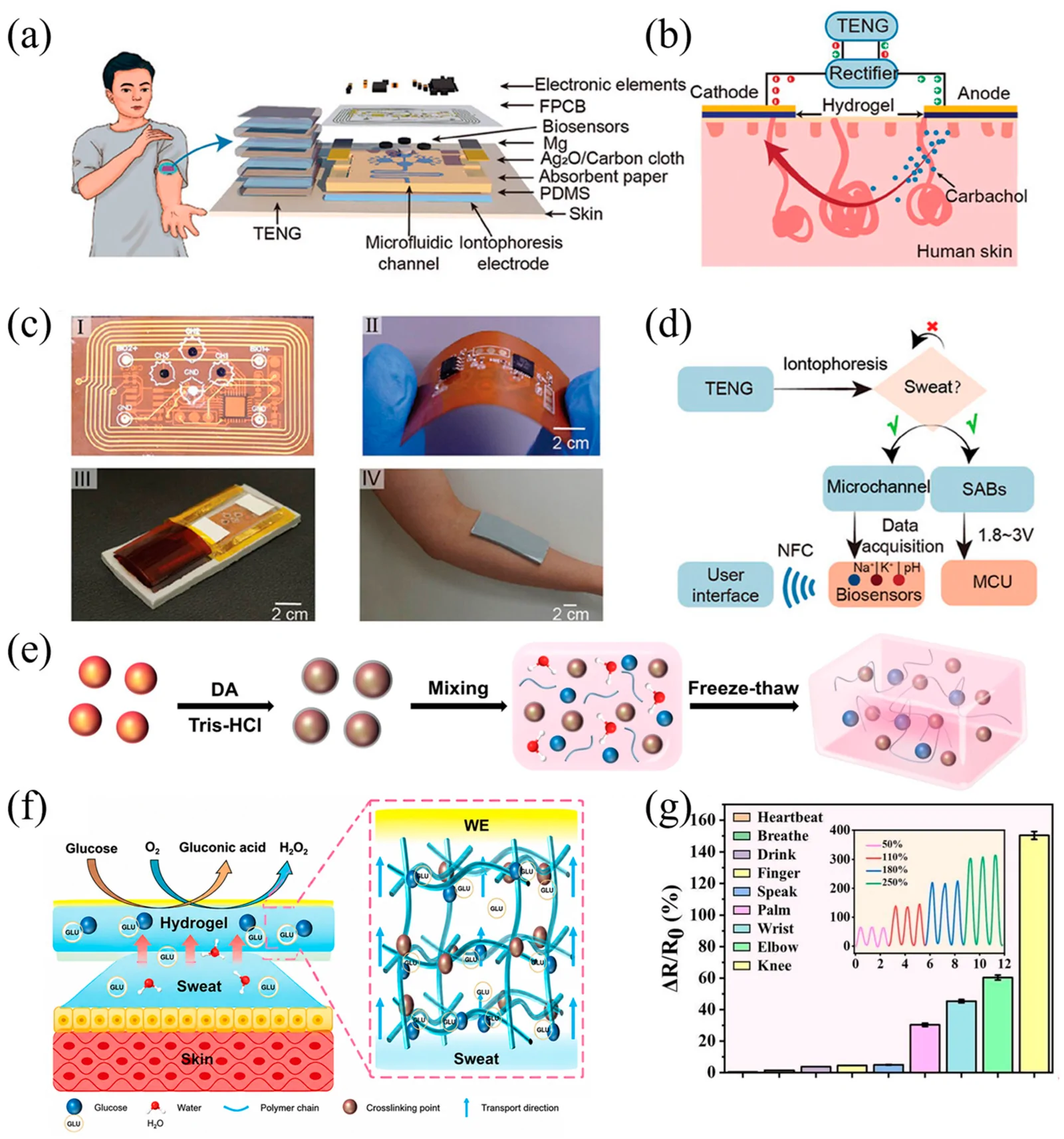
Design and operation of the self-powered monitoring system. (**a**) Schematic of the wearable SEMS for on-demand sweat analysis in sedentary users; (**b**) TENG-assisted sweat stimulation; (**c**) photographs of the SEMS device: (I) flexible microcircuit integrated with biosensors, (II) bending capability of the FPCB, (III) PDMS-encapsulated TENG and circuit, and (IV) conformal attachment to the skin; (**d**) operational workflow of the SEMS platform. Reprinted/adapted with permission from Ref. [55]. Copyright 2024, Wiley Online Library. (**e**) Preparation of AuNPs@PDA and AuNPs@PDA–PVA hydrogels; (**f**) sweat glucose-sensing mechanism; (**g**) motion signals recorded from different body regions. Reprinted/adapted with permission from Ref. [57]. Copyright 2023, Elsevier.

For example, Xu et al. reported a TENG-enabled, self-powered sweat monitoring system that integrated sweat induction, microfluidic transport, biosensing, and NFC-based wireless readout within a flexible wearable platform (Figure 11a) [55]. The TENG powered iontophoresis to stimulate sweat secretion under sedentary or low-sweating conditions (Figure 11b). Photographs in Figure 11c show the biosensor-integrated flexible circuit, its bending capability, PDMS encapsulation, and conformal attachment to the skin. The complete workflow, including sweat stimulation, microfluidic collection, biochemical analysis, signal processing, and wireless transmission, is summarized in Figure 11d. This integrated design reduces reliance on naturally induced sweating and supports continuous, noninvasive monitoring under low-activity conditions.

Another representative work by Hou et al. demonstrated a mussel-inspired, self-healing PVA/AuNPs@PDA hydrogel for simultaneous motion and sweat-glucose sensing [57]. The preparation of AuNPs@PDA and the resulting composite hydrogel is illustrated in Figure 11e. After incorporating glucose oxidase, enzymatic oxidation of glucose generated concentration-dependent electrochemical signals, enabling detection over 1.0–200.0 μmol L^−1^ with a limit of 0.9 μmol L^−1^ (Figure 11f). Resistance changes under deformation further allowed the hydrogel to distinguish motions from different body regions (Figure 11g). Although not a conventional TENG, this system provides a useful model for integrating soft, self-healing interfaces with biochemical recognition modules in self-powered sweat sensors.

However, sweat-based TENSor still faces several clear limitations. For example, sweat secretion rate is strongly affected by individual variability, ambient temperature and humidity, and exercise intensity, making it difficult for the sensing signal to fully correspond to the actual physiological concentration. In addition, low-abundance biomarkers in sweat, such as glucose and cortisol, are susceptible to interference from skin contamination and sweat evaporation. Because sweat composition does not show a simple linear correlation with blood biomarkers, more reliable physiological calibration models are still required before sweat-based TENS can be used for early disease screening.

### 4.2. Tear-Based TENSor

The clinical appeal of tear sensing lies in its association with both ocular surface diseases and systemic metabolic conditions. Tears contain glucose, proteins, inflammatory factors, electrolytes, and indicators related to tear film stability, which can provide useful information for the auxiliary diagnosis of diabetes, dry eye disease, keratitis, and ocular inflammation. Compared with sweat, tears are available in much smaller volumes and exhibit lower flow rates, but they show stronger relevance to local ocular pathology, making them particularly suitable for early monitoring of eye-related diseases.

**Figure 12:**
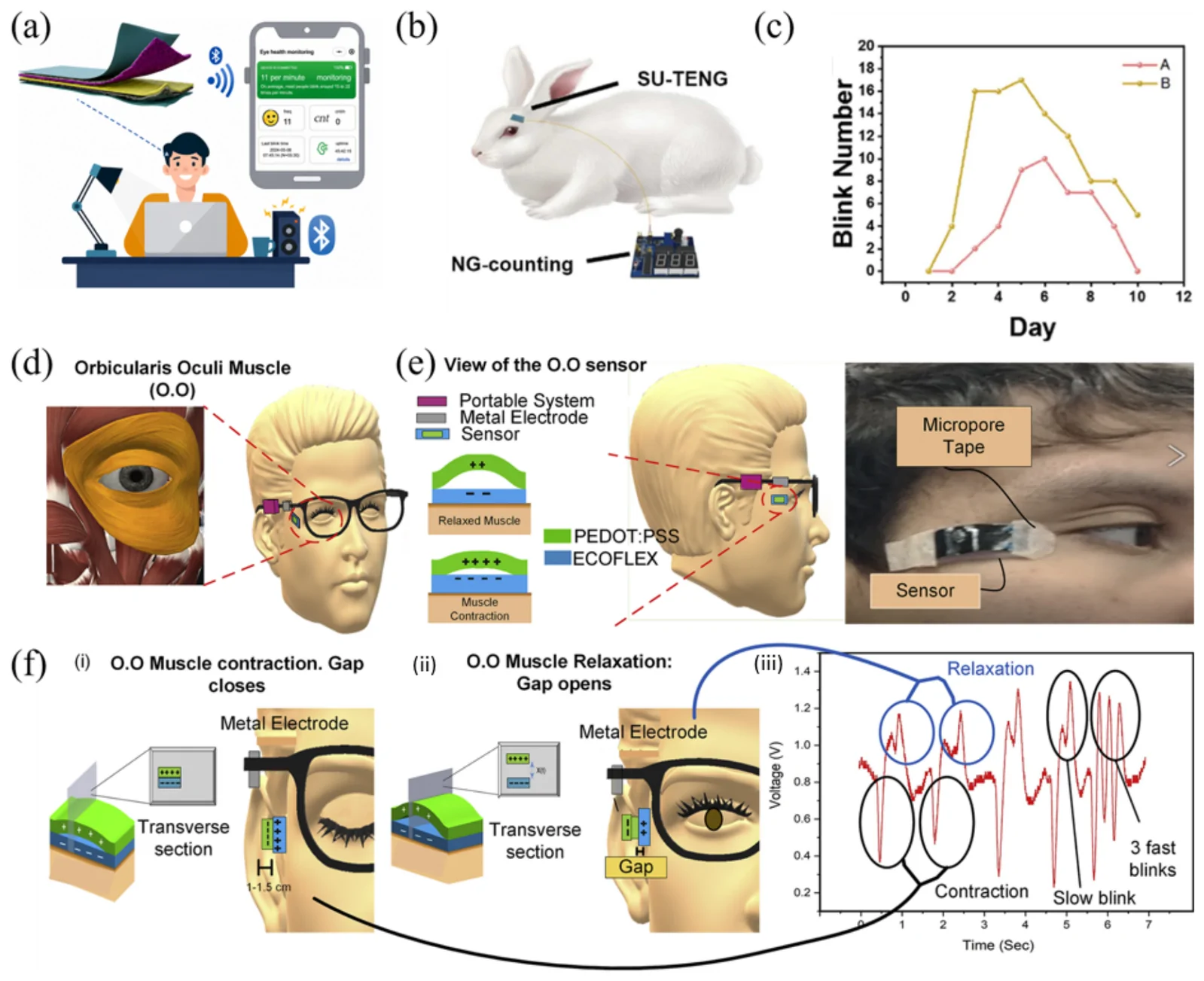
Triboelectric system for dry-eye monitoring and early warning: (**a**) System architecture integrating a stretchable blink sensor with counting, display, and human–machine interaction modules for visual and voice alerts; (**b**) schematic of the rabbit dry-eye model; (**c**) blink frequency recorded over 5 min after atropine sulfate instillation. Reprinted/adapted with permission from Ref. [98]. Copyright 2024, Elsevier. Eye-motion sensor design and response: (**d**) Location of the orbicularis oculi muscle; (**e**) sensor configuration and placement; (**f**) sensor deformation during eye closure and reopening: (i) Sensor deformation during eye closure (muscle contraction and gap closing), (ii) sensor recovery during eye reopening (muscle relaxation and gap opening), (iii) representative signals for fast and slow blinks, with representative signals for fast and slow blinks. Reprinted/adapted with permission from Ref. [99]. Copyright 2020, Elsevier.

The role of TENGs in tear sensing is not limited to power supply; rather, they can generate signals from blinking, eyelid motion, and tear wetting processes. Eye patch-based devices can produce periodic triboelectric outputs through eyelid movement, enabling the monitoring of blink frequency, eye fatigue, and tear film changes. Contact lens-based TENGs further place the sensing interface directly in the ocular surface environment, offering the potential for continuous detection of tear glucose or inflammatory factors. For example, Xiong et al. developed a self-powered, stretchable, and ultrathin triboelectric system for dry-eye monitoring and early warning [98]. The platform integrated blink sensing with signal counting, visual display, and human–machine feedback modules (Figure 12a). Its performance was evaluated in an atropine-induced rabbit dry-eye model (Figure 12b), where changes in blink frequency were recorded over 5 min throughout the experimental period (Figure 12c). The system enabled sensitive recognition of blinking behavior and timely visual or voice alerts, supporting noninvasive ocular health monitoring.

In addition, Vera Anaya et al. reported a self-powered eye-motion sensor based on triboelectric coupling and near-field electrostatic induction [99]. The device was positioned over the orbicularis oculi muscle, whose contraction and relaxation during blinking provided the mechanical stimulus for signal generation (Figure 12d). Its configuration and on-skin placement are shown in Figure 12e. Eye closure compressed the sensing gap, whereas reopening restored the initial spacing, producing distinct electrical responses for muscle contraction and relaxation as well as for fast and slow blinks (Figure 12f). The sensor further supported wireless eye-motion monitoring, hands-free control, and fatigue assessment. Nevertheless, translation of ocular TENGs remains challenging because devices must combine softness, low irritation, breathability, and long-term biocompatibility without disturbing tear-film stability or consuming the limited tear volume.

### 4.3. Saliva-Related TENSor

Saliva is one of the most accessible biofluids in the oral environment and contains electrolytes, proteins, enzymes, hormones, and microbiota-related molecules, which can provide information on oral health, stress status, and certain systemic metabolic conditions. Typical sensing targets include pH, α-amylase, cortisol, and biomarkers associated with oral infection. However, compared with sweat- and tear-based systems, saliva-related triboelectric sensing is still less developed for direct quantitative analysis of salivary biochemical components. Existing studies more commonly exploit the oral cavity as a saliva-containing and mechanically active environment, where speaking, chewing, biting, swallowing, and tongue movement provide continuous mechanical stimuli for triboelectric signal generation. In this sense, current devices are better described as saliva-related oral triboelectric sensors rather than conventional saliva biochemical sensors.

In this scenario, the main advantage of TENGs lies in self-powered sensing of oral biomechanical behaviors. By integrating flexible, biodegradable, or biocompatible triboelectric materials into dental braces, oral patches, bite-responsive structures, or swallowing rehabilitation devices, oral motions can be converted into electrical signals for oral healthcare monitoring, chewing behavior assessment, and swallowing function evaluation. Although these signals cannot yet be directly equated with changes in salivary pH, enzymatic activity, or hormone concentration, they demonstrate the feasibility of TENG operation in the humid, ionic, and mechanically dynamic oral environment, providing a basis for future integration with salivary biochemical recognition modules.

**Figure 13:**
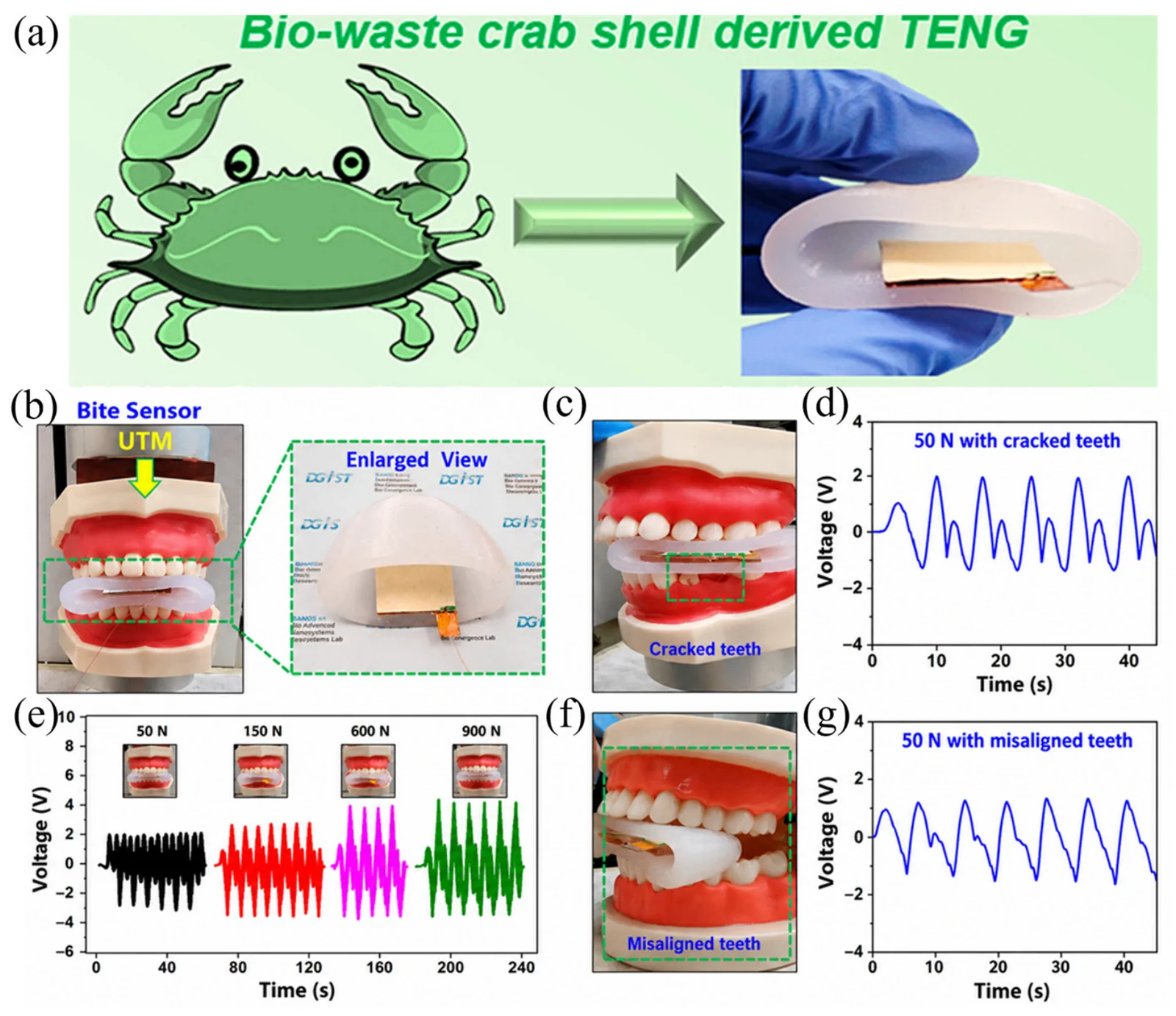
TENG-based oral bite sensing. (**a**) Crab shell-derived TENG and encapsulated bite sensor; (**b**) experimental setup and enlarged sensor view; (**c**,**d**) cracked-tooth model and voltage response at 50 N; (**e**) output signals under bite forces of 50–900 N, the black, red, pink, and green curves represent the output voltage of 50 N, 150 N, 600 N, and 900 N, respectively.; (**f**,**g**) misaligned-tooth model and corresponding voltage response at 50 N. Reprinted/adapted with permission from Ref. [100]. Copyright 2023, American Chemical Society.

For example, Panda et al. developed a sustainable single-electrode TENG using biowaste-derived cellulose, chitosan, and gelatin for oral healthcare monitoring [100]. Among the tested composites, the crab shell-derived PVA/chitosan material produced the highest output and was encapsulated as a flexible bite sensor (Figure 13a). The sensor was evaluated using a dental model and a universal testing machine (Figure 13b), where distinct voltage patterns were obtained for cracked teeth under a 50 N bite force (Figure 13c,d). Its output also increased with applied forces from 50 to 900 N (Figure 13e). Misalignment between the upper and lower teeth generated a different characteristic waveform (Figure 13f,g), demonstrating the potential of biodegradable TENGs for battery-free bite-force measurement and dental-abnormality screening. Meanwhile, Yun et al. proposed a self-powered triboelectric sensor for swallowing rehabilitation, in which tongue pressing, licking, and holding motions were converted into electrical signals [101]. This work further highlights the feasibility of using oral biomechanical activities as natural driving sources for TENG-based intraoral sensing.

The bottlenecks of saliva-related oral TENGs mainly arise from the complexity of the oral environment. Saliva composition is readily affected by diet, oral hygiene, circadian rhythm, and microbial communities, leading to substantial fluctuations in the sensing background. Meanwhile, intraoral devices must also address biofouling, mechanical wear, accidental swallowing risks, and long-term wearing comfort. At the current stage, saliva-related TENGs are therefore more suitable for short-term and event-driven sensing scenarios, such as oral health monitoring, chewing behavior analysis, swallowing rehabilitation, or stress-related behavioral assessment, rather than serving as direct substitutes for clinical blood tests or mature saliva biochemical assays.

### 4.4. Urine-Based TENSor

Urine analysis has a relatively mature clinical basis in disease screening and health assessment, particularly for the preliminary evaluation of urinary system disorders, renal dysfunction, and metabolic abnormalities. Urinary biomarkers such as glucose, proteins, uric acid, ketone bodies, pH, and ion concentrations are of important diagnostic value. Compared with sweat, saliva, and tears, urine is available in larger volumes, can be collected more conveniently, and has already been widely used in home testing and routine clinical analysis. These features make urine a promising target for translational applications of triboelectric biofluid sensing.

**Figure 14:**
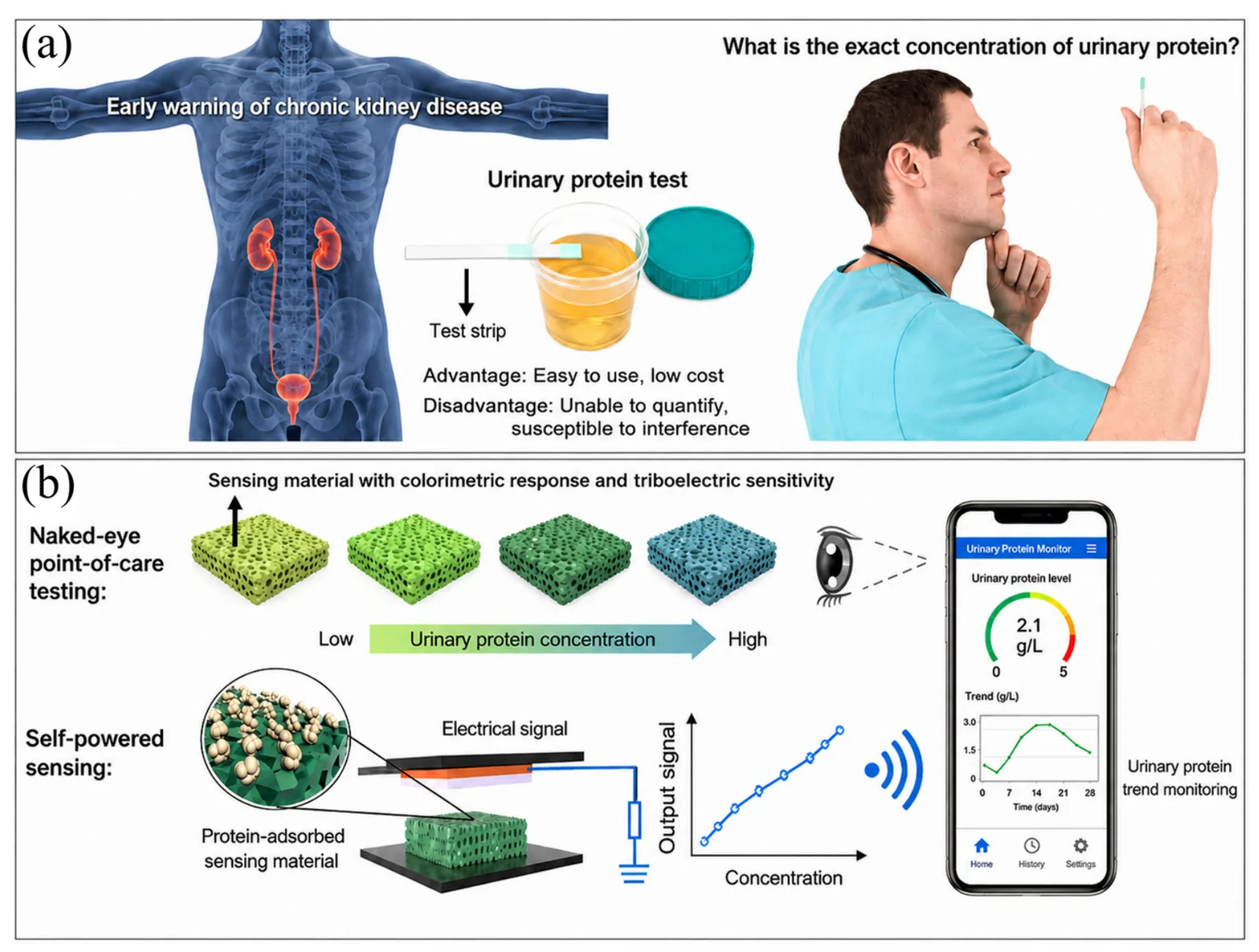
Urinary protein screening strategies. (**a**) Working principle and limitations of conventional urine test strips; (**b**) workflow for visual screening and triboelectric quantitative analysis using a color-responsive sensing material. Reprinted/adapted with permission from Ref. [102]. Copyright 2025, American Chemical Society.

In TENG systems, urine is particularly suitable for solid–liquid triboelectric sensing. When continuous urine flow or urine droplets impact a functional surface, the contact, wetting, and separation processes at the liquid–solid interface can generate electrical signals. These signals are not only related to flow rate, droplet volume, and surface wettability, but are also affected by urinary ion concentration and chemical composition. Therefore, urine-based TENGs can serve not only as self-powered sensing platforms but also as tools for monitoring urination behavior, urinary flow rate, and sample transport status, providing new device strategies for smart toilets, home urinalysis, and portable urine testing. For example, Zhou et al. developed a self-powered platform for urinary protein screening using a sensing material with both colorimetric and triboelectric responses [102]. Conventional urine test strips are simple and inexpensive but provide limited quantitative accuracy and are vulnerable to interference (Figure 14a). The proposed material exhibited concentration-dependent color changes for rapid visual screening, while its triboelectric output enabled quantitative analysis and trend monitoring through a mobile interface (Figure 14b). This dual-mode strategy offers a practical route toward home-based urinary protein monitoring and early warning of chronic kidney disease.

Beyond urinary protein analysis, Baraily et al. proposed a PVDF-HFP/WS_2_ nanosheet-based droplet TENG for the possible detection of Na^+^/K^+^ ion concentrations [103]. The device generated electrical outputs through liquid–solid contact during droplet impact, while changes in ionic concentration altered the charge transfer and ion adsorption behavior at the interface. By further testing artificial urine, the system showed concentration-dependent responses to urinary Na^+^ ions, demonstrating the potential of droplet-based TENGs for body-fluid ion analysis.

The advantages of urine-based TENSor lie in abundant sample availability, well-defined testing scenarios, and easy integration with home diagnostic devices or smart sanitation platforms. However, salts, proteins, and metabolites in urine can readily deposit on device surfaces, causing biofouling, interfacial contamination, and signal attenuation. Individual differences in water intake, diet, medication use, and sampling time can also significantly affect urine concentration and the sensing background. For early disease screening, urine-based TENSor needs to integrate multi-parameter analysis, antifouling interfaces, replaceable sensing modules, and standardized calibration methods to improve the reliability and clinical interpretability of the detection results.

### 4.5. Interstitial Fluid-Based TENSor

Interstitial fluid is an abundant extracellular fluid in the human body and continuously exchanges substances with blood through capillaries. Therefore, its composition can reflect metabolic information in the blood to a certain extent. Compared with sweat and saliva, interstitial fluid shows stronger correlations with biomarkers such as glucose, lactate, ions, and drug concentrations. Compared with blood, its collection can be made minimally invasive through technologies such as microneedles. Therefore, interstitial fluid is considered one of the most promising biofluids for continuous metabolic monitoring.

**Figure 15:**
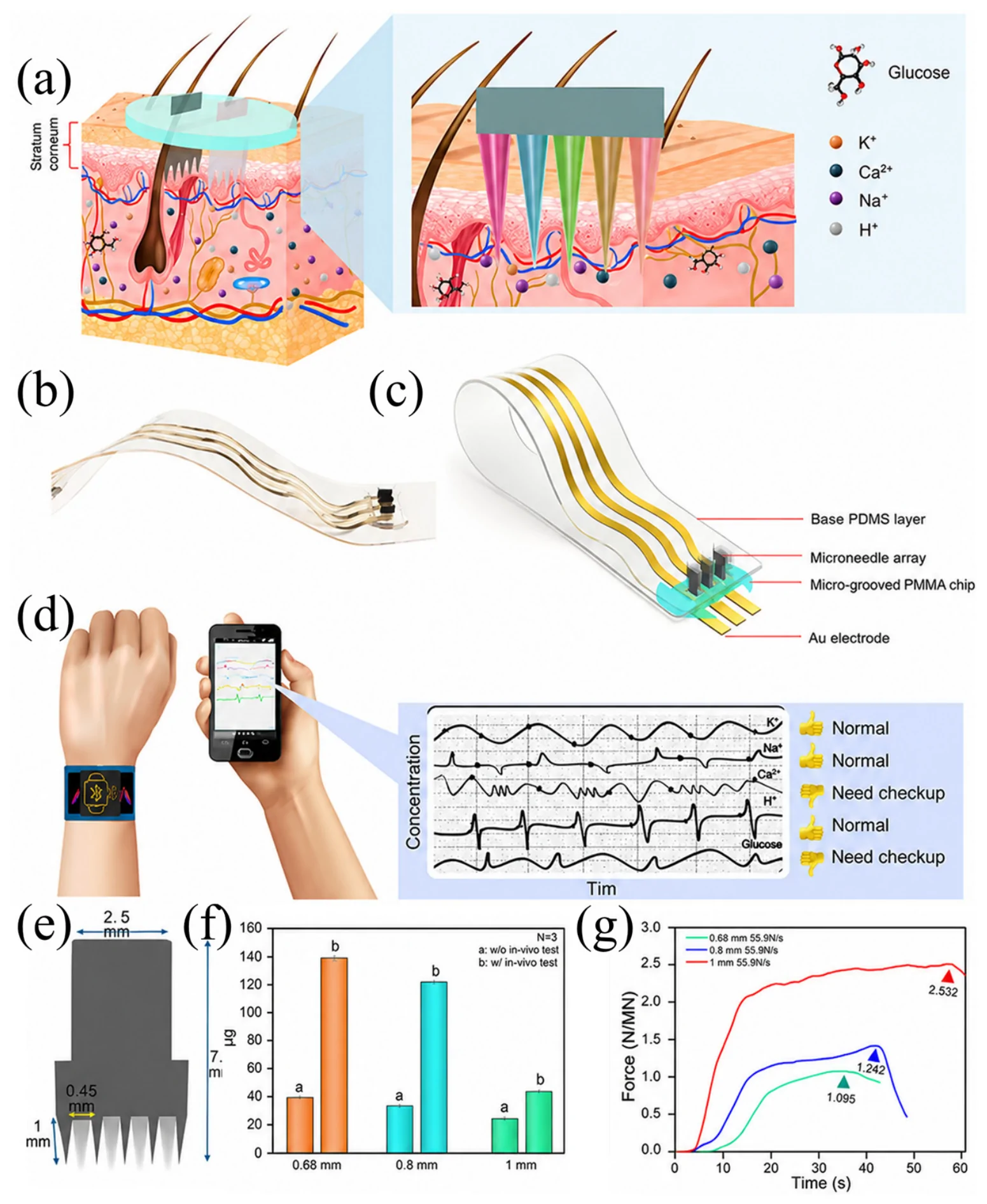
Wearable microneedle biosensor for continuous metabolic monitoring. (**a**) ISF extraction and real-time detection of glucose, K^+^, Ca^2+^, Na^+^, and H^+^; (**b**) photograph of the flexible microneedle device; (**c**) multilayer structure comprising PDMS, Au electrodes, a microgroove PMMA chip, and a microneedle array; (**d**) wrist-mounted sensing with wireless smartphone readout; (**e**) microneedle dimensions; (**f**) protein content before and after in vivo use for different needle lengths (*n* = 5); (**g**) force–displacement responses of microneedles with different geometries. Reprinted/adapted with permission from Ref. [104]. Copyright 2026, Wiley Online Library.

The integration of TENGs with interstitial fluid sensing usually relies on microneedle arrays, flexible electrodes, and skin-adherent structures. Microneedles penetrate the stratum corneum and extract trace amounts of interstitial fluid, while TENGs can harvest energy from human motion to power sensing modules or serve as sensing units for pressure, adhesion status, and fluid transport processes. For example, Pal et al. reported an energy-autonomous microneedle platform for continuous biomarker monitoring in interstitial fluid [104]. The microneedle array penetrated the stratum corneum and simultaneously detected Na^+^, K^+^, Ca^2+^, pH, and glucose (Figure 15a). Its flexible form and multilayer architecture, integrating Au electrodes, a microgroove PMMA chip, and a PDMS substrate, are shown in Figure 15b,c. The wrist-mounted device transmitted real-time biomarker profiles to a smartphone for physiological assessment (Figure 15d). Microneedle dimensions were optimized to balance sampling efficiency and tissue penetration (Figure 15e), while protein accumulation after in vivo use and the mechanical strength of different microneedle geometries were evaluated in Figure 15f,g. A hybrid TENG–electromagnetic generator harvested body-motion energy to support wireless data transmission, enabling continuous monitoring during daily activity and exercise.

In addition, Zhang et al. reported a self-powered wearable reverse iontophoresis system driven by a TENG for interstitial fluid lactate monitoring [105]. The TENG harvested biomechanical energy to drive transdermal lactate extraction through reverse iontophoresis, followed by visual detection using a colorimetric lactate sensor. In vitro and in vivo tests confirmed efficient lactate extraction and exercise-induced lactate monitoring without obvious skin damage, demonstrating the potential of TENG-driven systems for noninvasive and continuous metabolic analysis. For early diagnosis, the clinical significance of interstitial fluid-based TENSor does not lie in replacing blood tests, but in continuously capturing trends in metabolic fluctuations, such as postprandial glucose changes, exercise-induced lactate accumulation, or dynamic variations in drug concentrations.

### 4.6. Blood TENSor

Blood remains the most information-rich biofluid in disease diagnosis and has the most mature framework for clinical interpretation. It contains cells, proteins, metabolites, inflammatory factors, and pathogen-related biomarkers, providing diagnostic information for diabetes, cardiovascular diseases, infections, tumors, immune abnormalities, and other diseases. Compared with sweat, tears, saliva, and urine, blood sensing offers clear advantages in terms of higher biomarker abundance, well-established clinical relevance, and relatively mature diagnostic standards. Its limitations, however, mainly arise from invasive sampling, complex sample pretreatment, and reliance on professional instruments and operating conditions.

**Figure 16:**
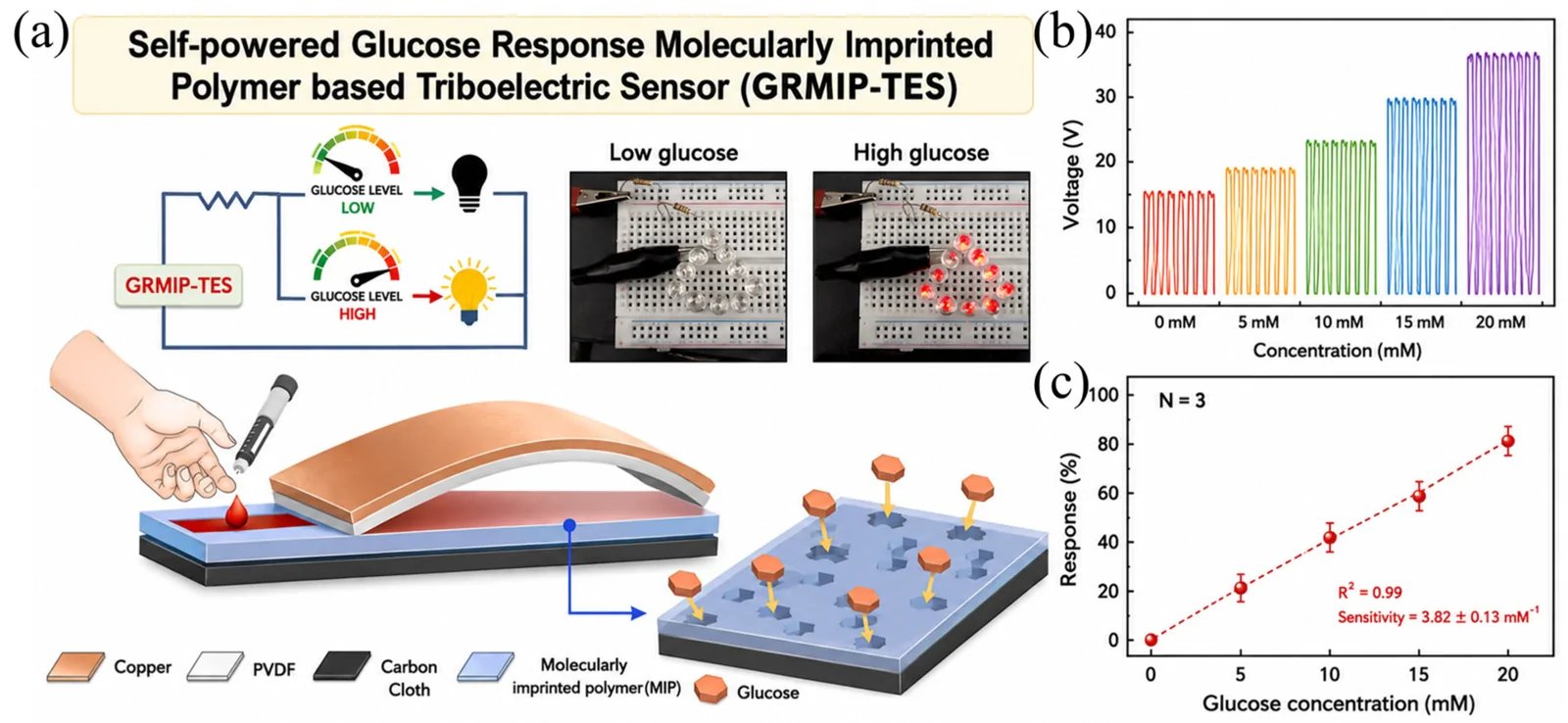
Design and glucose-sensing performance of the self-powered GRMIP-TES. (**a**) Device structure, molecularly imprinted glucose recognition, and LED-based concentration indication; (**b**) triboelectric voltage responses at glucose concentrations of 0–20 mM; (**c**) calibration curve of the concentration-dependent sensor response (*n* = 3). Reprinted/adapted with permission from Ref. [106]. Copyright 2024, Elsevier.

In triboelectric blood sensing systems, the role of TENGs has gradually expanded from simple energy harvesting to signal transduction and sample analysis. On the one hand, TENGs can harvest energy from human motion or fluid flow to power microfluidic chips, electrochemical sensors, signal-processing circuits, and wireless transmission modules, thereby promoting the miniaturization, portability, and point-of-care implementation of blood-testing devices. On the other hand, blood droplet impact, blood-flow transport, and solid–liquid interfacial contact within microchannels can themselves induce triboelectric outputs, allowing ionic concentration, electrical conductivity, glucose level, or interfacial adsorption behavior in blood to be converted into readable electrical signals. Owing to this dual capability of energy supply and sensing response, TENGs provide a new strategy for constructing low-sample-volume, rapid-response, and self-powered blood analysis platforms. For example, Chang et al. developed an enzyme-free, self-powered glucose sensor based on a glucose-responsive molecularly imprinted polymer (GRMIP-TES) [106]. The device integrated a glucose-imprinted poly (3-aminophenylboronic acid) recognition layer with a triboelectric structure and enabled LED-based indication of elevated glucose levels (Figure 16a). Selective glucose adsorption altered the surface charge characteristics of the imprinted layer, producing progressively higher voltage outputs over 0–20 mM glucose (Figure 16b). The sensor exhibited a near-linear concentration response with a sensitivity of 3.82 ± 0.13 Mm^−1^ (Figure 16c), together with good selectivity, stability, and reusability, supporting its potential for self-powered glucose monitoring.

In another representative study, Luo et al. reported a portable milli-fluidic nanogenerator lab-on-a-chip device for low-frequency blood electrical conductivity monitoring [107]. In this system, blood or plasma served as the conductive medium in the built-in TENG, and the generated voltage patterns were used to estimate sample conductivity. Tests with simulated body fluid and human blood plasma confirmed its ability to detect conductivity variations associated with electrolyte concentration changes. Combined with artificial intelligence analysis and a 3D-printed disposable design, this work offers a simple and self-powered point-of-care strategy for rapid blood conductivity assessment.

However, blood is not the biofluid most readily compatible with TENSor. Its high viscosity, complex protein composition, and tendency toward coagulation can readily cause microchannel clogging, interfacial contamination, and unstable signal outputs. In addition, blood samples usually require anticoagulation, separation, or dilution before analysis, which weakens the intrinsic advantages of TENG systems in simplicity, portability, and self-powered operation. Therefore, blood-based TENSor is more suitable for low-power POCT platforms, microvolume sample analysis, or coupling with established electrochemical detection methods, rather than overemphasizing the concept of fully continuous and noninvasive monitoring.

### 4.7. Wound Exudate Detection

Wound exudate is a highly valuable yet long-underestimated diagnostic medium in chronic wound management. It contains information related to pH, inflammatory factors, immune proteins, bacterial metabolites, and toxins, and its changes can reflect infection, inflammatory responses, and tissue repair status. For diabetic foot ulcers, burns, and long-term nonhealing wounds, wound exudate monitoring is important not only for diagnosis but also for guiding the timing of treatment and the intensity of intervention.

**Figure 17:**
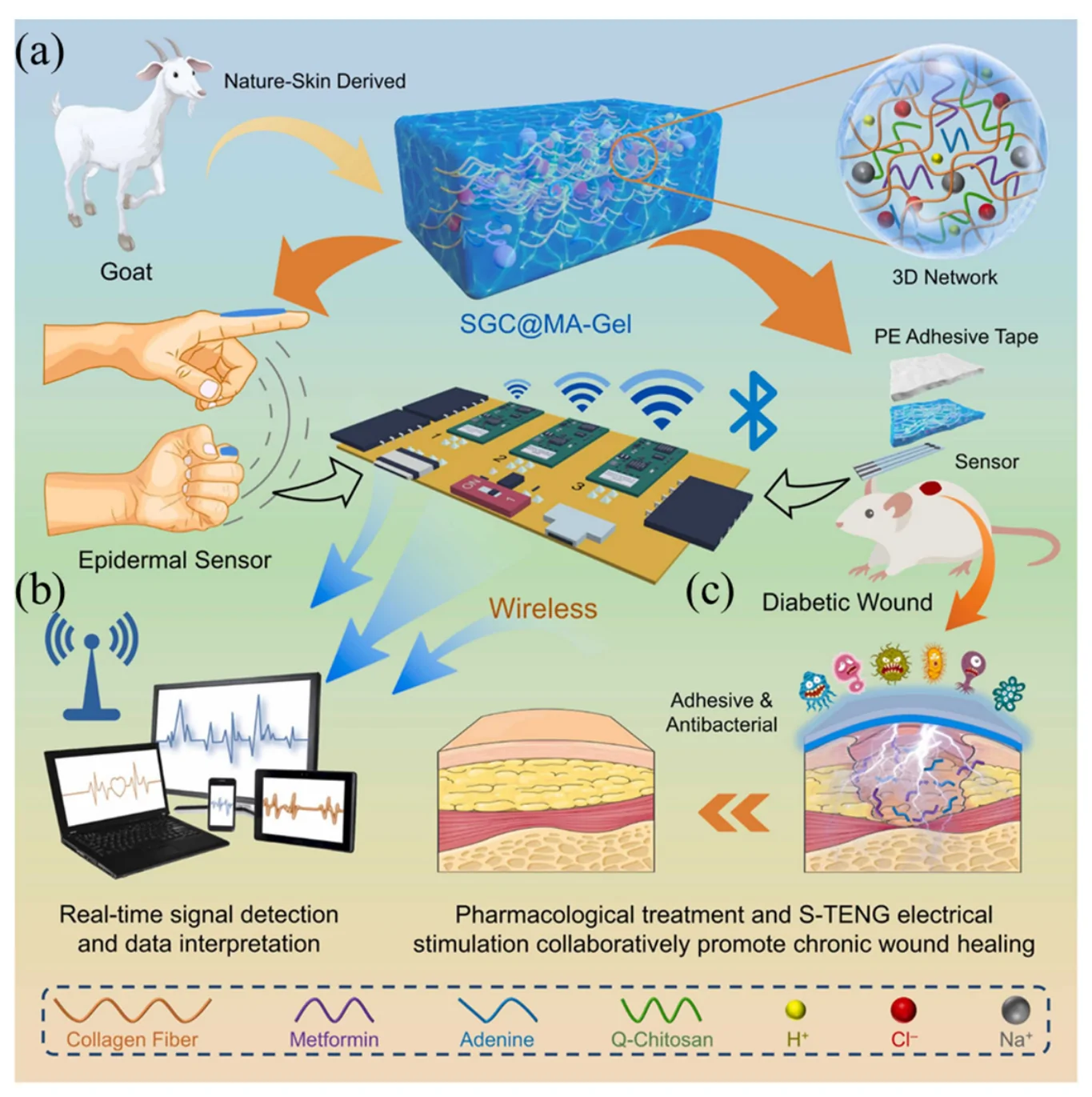
SGC@MA-Gel-based bioelectronic system for diabetic wound monitoring and treatment. (**a**) Material design and integration of the hydrogel-based epidermal sensor and S-TENG; (**b**) wireless signal acquisition and real-time data analysis; (**c**) combined pharmacological and electrical stimulation for diabetic wound healing. Reprinted/adapted with permission from Ref. [56]. Copyright 2023, Elsevier.

The advantages of TENGs in wound exudate detection are clearly distinct from those in other biofluid sensing scenarios. Since wound dressings are required to remain attached to the skin surface for prolonged periods, TENGs can be naturally embedded into dressing structures. Electrical signals can be generated through human motion, dressing deformation, exudate flow, or droplet contact, enabling wound status monitoring. Meanwhile, local electrical stimulation produced by TENGs may promote cell migration, angiogenesis, and tissue regeneration, and can synergize with antibacterial materials to suppress infection. Therefore, wound-related TENGs are not merely sensors, but are closer to theragnostic smart dressings that integrate diagnosis and therapy. For example, Bai et al. developed a battery-free, wireless bioelectronic platform based on a skin-derived SGC@MA-Gel for chronic wound monitoring and treatment [56]. The hydrogel was integrated into an epidermal sensor and a single-electrode TENG for motion sensing and self-powered electrical stimulation (Figure 17a). Wound biomarkers, including glucose, uric acid, and pH, were monitored and transmitted wirelessly for real-time analysis (Figure 17b). The adhesive and antibacterial dressing further combined drug therapy with TENG-driven stimulation to accelerate diabetic wound healing (Figure 17c), demonstrating an integrated strategy for wound assessment and treatment.

As another example, Wang et al. integrated bilayer microneedle dressings with a TENG for infected wound management [108]. In this system, antibiotic-loaded dissolvable microneedles provided sustained drug release, while TENG-generated electrical stimulation was delivered to the wound through conductive stainless-steel microneedles. The CNT/Ag-coated microneedles further enabled electrochemical detection of hydrogen peroxide and uric acid, confirming the potential of MN-TENG systems for combining infection control, wound monitoring, and active tissue repair.

Nevertheless, the wound exudate environment is complex and highly dynamic. The amount of exudate fluctuates with different wound-healing stages, while necrotic tissue, blood, bacterial biofilms, and residual drugs may all interfere with detection results. More importantly, wound dressings must simultaneously satisfy requirements for exudate absorption, breathability, antibacterial activity, low adhesion, replaceability, and biocompatibility. To provide a broader comparison across the biofluid systems discussed above, representative BF-TENG platforms are summarized in Table 1 according to their materials, biofluid types, electrical outputs, technological roles, and sample sources.

## 5. Conclusions and Perspectives

Triboelectric nanogenerators provide a self-powered, flexible, and continuous sensing strategy for biofluid analysis. In recent years, BF-TENG sensing systems using sweat, tears, saliva, urine, interstitial fluid, blood, and wound exudate have been widely explored, showing considerable potential in metabolic monitoring, infection assessment, chronic wound management, and personalized healthcare. Compared with conventional analytical methods, TENG devices can convert human motion, droplet contact, and biofluid flow into electrical signals, thereby reducing reliance on external power supplies and complex analytical instruments.

Nevertheless, BF-TENGs still remain some distance from true early disease diagnosis. Biofluid compositions are highly complex, and biomarker concentrations are strongly affected by individual differences, environmental conditions, and physiological states. Meanwhile, TENG outputs are susceptible to interference from contact pressure, motion frequency, liquid flow rate, ionic strength, and interfacial contamination. Future research should therefore move beyond the pursuit of high output voltage or power density and place greater emphasis on signal accuracy, long-term stability, biosafety, wearing comfort, sustainable design, and clinical translation. Only when TENG signals can stably and reproducibly reflect real physiological changes can this technology move from laboratory prototypes toward practical health management and early disease screening.

### 5.1. Early Screening and Clinical Validity

Early disease screening represents one of the most clinically promising directions for BF-TENGs. Biofluids such as sweat, tears, saliva, urine, interstitial fluid, and wound exudate contain electrolytes, metabolites, hormones, inflammatory factors, and infection-related biomarkers, providing an important basis for noninvasive or minimally invasive health monitoring. Compared with single-point clinical testing, BF-TENGs are more suitable for continuously capturing dynamic physiological changes, such as electrolyte imbalance, lactate accumulation, glucose fluctuation, stress response, urinary metabolic abnormalities, and wound infection progression.

It should be emphasized that most current BF-TENGs are more suitable as tools for early risk screening and trend monitoring rather than direct clinical diagnosis. Evidence obtained only from artificial biofluids, nonphysiological concentration ranges, or benchtop experiments cannot alone establish clinical screening validity. Clinical validation requires paired comparisons with standard clinical assays to determine concentration correlations, interindividual variability, and the time lag between biomarker changes in noninvasive biofluids and corresponding clinical reference measurements. Because biofluid biomarker levels are often affected by diet, exercise, circadian rhythm, and individual variability, a single signal is rarely sufficient to accurately identify a specific disease state. Future BF-TENGs should therefore evolve from simple signal-response devices into integrated multiparameter analytical platforms. By combining microfluidic sampling, electrochemical detection, colorimetric recognition, temperature and humidity compensation, wireless transmission, and data algorithms, the reliability and clinical interpretability of early screening results can be further improved.

Different biofluids should also be matched with different screening scenarios. Sweat is more suitable for monitoring exercise metabolism and dehydration; tears are promising for ocular disease assessment and glucose-related monitoring; saliva can be used for stress evaluation and oral health analysis; urine is suitable for preliminary screening of metabolic and urinary system disorders; interstitial fluid is particularly attractive for continuous metabolic monitoring; and wound exudate is more appropriate for early warning of chronic wound infection. Future device design should be driven by specific disease requirements rather than by the simple conceptual stacking of multiple biofluids and multiple biomarkers.

### 5.2. Stability and Accuracy

Stability and accuracy are central limitations that restrict the practical application of BF-TENGs. For conventional TENGs, output stability usually refers to whether voltage, current, or charge can be maintained after repeated operating cycles. In biofluid sensing, however, stability also involves controllable biofluid transport, resistance to interfacial contamination, signal drift, and whether the output variation truly originates from the target biomarker.

Ion concentration, pH, protein adsorption, and microbial contamination in biofluids can all influence charge transfer at the solid–liquid interface. For instance, high ionic strength may screen surface charges, protein deposition can alter surface wettability, and the complex components in blood and wound exudate may cause interfacial passivation or microchannel blockage. These factors can introduce nonspecific coupling between the TENG output and the target biomarker, thereby reducing detection accuracy. The kinetics of biofouling cannot be adequately captured by short-term cycling tests in simple buffer solutions. Continuous or repeated exposure to real biofluids should be used to quantify signal retention, baseline drift, fouling onset, and recovery after cleaning.

Future efforts should improve the stability of BF-TENGs from two aspects. First, microfluidic structures, hydrophilic–hydrophobic patterning, antifouling coatings, and replaceable sensing modules should be used to standardize biofluid collection and transport. Second, more reliable signal calibration methods are needed to distinguish the contributions of mechanical disturbance, liquid flow rate, ionic strength, and target molecule concentration to the output signal. Machine-learning models have been used to predict the behavior of multivariable chemical systems [109], and could similarly help separate mechanical interference from biomarker-dependent changes in BF-TENG outputs. For early disease screening, the key question is not whether the device can generate a high output, but whether the output can remain interpretable, reproducible, and comparable in complex human environments.

### 5.3. Biocompatibility and Biosafety

BF-TENGs usually need to directly contact the skin, ocular surface, oral cavity, wounds, or blood environment, making biocompatibility and biosafety unavoidable issues. Flexible polymers, natural macromolecules, hydrogels, metal nanoparticles, carbon materials, MXenes, enzymes, ionic liquids, and antibacterial components have all been used to improve sensing performance [110,111]. However, their safety during long-term contact with the human body requires systematic evaluation.

For skin-mounted sweat sensors, materials should avoid irritation, allergic reactions, and performance degradation after sweat exposure. For tear- and contact lens-based sensors, transparency, oxygen permeability, softness, and low irritation are particularly important. For oral sensors, devices must tolerate salivary enzymes, chewing friction, and complex microbial environments. For blood- and wound exudate-based sensors, protein adsorption, coagulation reactions, bacterial adhesion, and inflammatory risks must be carefully considered.

Some high-performance functional materials can enhance TENG outputs or provide antibacterial, catalytic, or conductive functions, but their long-term release behavior and metabolic safety remain unclear. Metal nanoparticles, for example, may raise concerns related to ion release, while two-dimensional materials may present potential cytotoxicity. Long-term safety assessments should also consider nanomaterial leaching, friction-induced wear debris, and the resulting tissue-specific toxicity and inflammatory responses, particularly at delicate interfaces such as the cornea, oral mucosa, and wound bed. Strong adhesive interfaces may also cause secondary damage during device removal. Future BF-TENGs should therefore shift from a performance-first design philosophy toward a coordinated design strategy that balances performance, safety, and application scenarios. Only with clearly defined safety boundaries can the diagnostic value of these devices become meaningful for clinical translation.

### 5.4. Mechanical and Durability

Most BF-TENG applications require long-term attachment or repeated use, making mechanical compliance and durability critical for user acceptance and stable operation. Mechanical performance should not be described only in terms of flexibility. Young’s modulus, tensile strength, elongation at break, bending stiffness, and fatigue resistance collectively determine conformal contact, effective contact area, and strain transfer at the triboelectric interface. For sweat, interstitial fluid, and wound exudate sensors, devices must remain in prolonged contact with the skin or wound surface and should therefore possess softness, breathability, conformability, and low modulus. Excessive stiffness or rigid nanomaterial loading may produce stress concentration, interfacial delamination, and tissue irritation, whereas excessive softening or swelling can alter the contact gap and pressure distribution, leading to unstable output. For tear and oral sensors, device size, thickness, edge structure, and mechanical stimulation must be strictly controlled; otherwise, normal blinking, vision, chewing, or speaking may be affected.

Tribological behavior is also directly related to charge generation and long-term reliability. Friction coefficient, surface roughness, and real contact area influence the initial contact-electrification efficiency, but repeated contact or sliding may damage micro/nanostructures, expose or release functional fillers, and cause electrode delamination or surface degradation. These changes can alter surface chemistry and charge density, producing gradual signal attenuation or baseline drift. In biofluid environments, water may provide interfacial lubrication, while salts, proteins, and deposited debris can dynamically change friction and wear behavior. Therefore, increasing surface roughness or friction does not necessarily improve long-term performance if it accelerates interfacial wear.

Durability is not limited to structural integrity after mechanical cycling. It also includes signal retention under humid, saline, enzymatic, frictional, and repeatedly bending conditions. Many TENG devices show good performance in dry laboratory environments, but their outputs may decrease significantly in real biofluid environments because of water absorption, swelling, interfacial contamination, or material fatigue. Future studies should therefore adopt testing conditions that more closely resemble real use scenarios, such as cyclic output measurements after sweat immersion, antifouling tests in simulated wound exudate, chewing-cycle tests for oral devices, blink-friction tests for ocular devices, and long-term stability evaluation under skin stretching. In addition to the number of cycles, applied force or strain, frequency, sliding distance, and environmental conditions should be reported. Mechanical property retention, friction coefficient, wear morphology, interfacial delamination, and electrical output retention before and after cycling would provide a more complete assessment of device durability.

At the same time, comfort and high performance often conflict with each other. Improving output usually requires stronger contact friction, increased surface roughness, or the introduction of rigid functional fillers, all of which may reduce softness and wearing comfort. Future designs should therefore use microstructural engineering, ultrathin encapsulation, elastic substrates, porous architectures, and low-modulus composites to achieve a balance between sensing performance and human comfort.

### 5.5. Biodegradation and Sustainability

With the development of disposable wearable sensors, smart dressings, and biofluid testing patches, the environmental burden of used devices is receiving increasing attention. Conventional TENGs often rely on nondegradable polymers, metal electrodes, and complex encapsulation materials. Although these components can provide high output and good stability, they may generate medical waste in large-scale health monitoring and home-testing applications. Biodegradability and sustainability will therefore become important directions for future BF-TENG development.

Biodegradable materials such as polylactic acid, polycaprolactone, poly(lactic-co-glycolic acid), silk fibroin, gelatin, chitosan, cellulose, and their derivatives provide opportunities for constructing green biofluid sensors. These materials generally show favorable biocompatibility and can be further optimized through structural design, surface modification, and composite reinforcement to meet the requirements of different biofluid environments. For example, short-term sweat patches and urine testing chips may prioritize environmental degradability; wound dressings should emphasize biosafe degradation and low irritation; and implanted or semi-implanted devices require more precise control over degradation time.

However, biodegradability does not automatically mean suitability for sensing applications. During degradation, materials may suffer from reduced mechanical strength, altered surface charge density, output attenuation, or accumulation of degradation products. Future research should therefore focus on controllable degradation rather than simply pursuing rapid degradation. An ideal BF-TENG should maintain stable performance during its service period and then gradually degrade or be safely removed after completing its monitoring or therapeutic function. Coordinating degradation rate, output stability, biosafety, and manufacturing cost remains a key challenge in this field.

### 5.6. Standardization and Clinical Translation

For BF-TENGs to move from laboratory prototypes toward early disease screening, a verifiable, comparable, and interpretable evaluation system must be established. At present, different studies vary greatly in device size, testing mode, simulated biofluid composition, external stimulation conditions, and data-processing methods, making direct performance comparison difficult. More unified testing standards are therefore needed to define how output performance, biosensing performance, and human-use conditions should be evaluated.

Given the diversity of BF-TENG configurations, standardization should define a common reporting framework while allowing application-specific test conditions. Artificial biofluid tests should disclose the full formulation, pH, conductivity, viscosity, temperature, and analyte concentration within physiological ranges. Mechanical inputs should be calibrated and reported in terms of force, displacement, frequency, contact area, and waveform; manual tapping or uncalibrated shakers are suitable only for qualitative demonstrations. Electrical characterization should report open-circuit voltage, short-circuit current, transferred charge, the tested load-resistance range, matched load resistance and corresponding power, effective device area, and the input impedance and sampling rate of the measurement instruments.

More importantly, the clinical value of BF-TENGs must be validated using real biofluid samples and benchmarked against standard analytical methods. Sweat, saliva, urine, interstitial fluid, blood, and wound exudate all show significant interindividual differences. Reliance solely on artificial biofluids or short-term in vitro experiments is insufficient to demonstrate early screening reliability. Future studies should gradually incorporate on-body testing, real-sample analysis, and clinical comparison experiments, and the results should be compared with established detection methods to clarify the specific role of BF-TENGs in risk screening, dynamic monitoring, or auxiliary diagnosis.

Clinical translation also requires compatibility with practical workflows. An ideal BF-TENG system should not only collect biofluids and generate signals, but also support simple operation, low-power readout, data recording, and result interpretation. Wireless transmission, smartphone-based readout, and algorithm-assisted analysis can serve as useful tools, but the ultimate goal should be to generate clear, reliable, and clinically understandable results for both physicians and patients. Only when BF-TENG outputs can be clearly correlated with specific physiological states or disease risks can this technology enter real clinical practice and home health management.

Commercial translation also depends on reproducible large-area manufacturing and stable product storage. Roll-to-roll coating or printing and screen-printing offer scalable alternatives to manual drop-casting and assembly, but film thickness, electrode alignment, batch yield, and device-to-device variation must be controlled. Hydrogels, enzymes, antibodies, and colorimetric reagents require hermetic packaging using barrier films, heat sealing, or lamination to limit moisture loss and oxygen exposure while preserving a defined biofluid-sampling window. Real-time and accelerated aging under ambient conditions should track TENG output, calibration response, reagent activity, adhesion, and seal integrity.

Overall, BF-TENGs hold broad promise for early disease screening, continuous health monitoring, and intelligent diagnosis and therapy. Their true value, however, depends on whether they can bridge the gap among material performance, signal reliability, biosafety, and clinical interpretability. Future research should shift from demonstrating that a device can work to proving that it is reliable, useful, and safe in real human environments. Through the coordinated development of material design, interfacial engineering, microfluidic management, multimodal sensing, and clinical standardization, BF-TENGs are expected to become an important component of next-generation self-powered bioelectronic devices.

## Figures and Tables

**Table 1:** Comparison of representative BF-TENG systems in terms of materials, biofluid types, electrical outputs, technological roles, and sample sources.

Material	Biofluid	Electrical Output	Role	Source	Ref.
FEP/PI	Sweat	240 V/17 μA	Power supply	Human volunteers	[55]
PDMS/TPU/Ag/PU	Tear	16 V/70–120 nA	Biomechanical sensing	Rabbits/Volunteers	[98]
Ecoflex/PEDOT:PSS–WPU/Cu	Tear	300–600 mV	Biomechanical sensing	On-body test	[99]
PVA/chitosan–rice paper/Ag/Ecoflex	Saliva	20 V/200 nA/12 nC	Biomechanical sensing	Dummy teeth model	[100]
RIE-PTFE/Cu/PET	Saliva	24.12 V/1.056 μA	Biomechanical sensing	On-body test	[101]
PDMS/PTFE/Cu	Urine	20 V	Biofluid sensing	Artificial urine	[102]
Nylon/PTFE/PET–Al	Interstitial fluid	400 V/5 μA	Power supply	Healthy volunteers	[104]
Silicone/conductive fabric	Interstitial fluid	400 V/30 μA	Power supply	In vitro/Mouse model	[105]
PVDF/poly(3-APBA)-carbon cloth/Cu	Blood	14–36 V	Biofluid sensing	Glucose/PBS solutions	[106]
PMMA/PTFE/Cu	Blood	7–10 V	Biofluid sensing	Clinical sample	[107]
PET/PTFE/Cu	Wound exudate	30 V/9 μA/10 nC	Treatment/Power supply	Artificial samples/Mice	[108]

## Data Availability

No new data were created or analyzed in this study. Data sharing is not applicable to this article.
